# Design of a Novel Diamine Biosensor to Guide the Engineering of *Escherichia coli* for Cadaverine Overproduction

**DOI:** 10.1002/advs.77720

**Published:** 2026-09-27

**Authors:** Jia Feng, Getao Liu, Ding Ma, Jiaxiang Yuan, Xinyi Zhang, Weihao Wang, Chen Ma, Yang Liao, Siyuan Gao, Lei Qin, Jiao Feng, Sheng Xu, Xin Wang, Kequan Chen

**Affiliations:** ^1^ State Key Laboratory of Materials‐Oriented Chemical Engineering College of Biotechnology and Pharmaceutical Engineering National Engineering Research Center for Biotechnology Nanjing Tech University Nanjing Jiangsu China; ^2^ Department of Chemical Engineering/Key Laboratory for Industrial Biocatalysis of Ministry of Education Tsinghua University Beijing China

**Keywords:** cadaverine biosensor, high‐throughput screening, responsive‐promoters, transcription factor, transcriptome

## Abstract

The development of in vivo biosensor systems represents a powerful strategy for engineering cell factories with desired production. Here, we reported a paradigm for the design, mechanistic elucidation, and optimization of the novel biosensor to detect cadaverine, an important polymer monomer. We identified a diamine‐responsive promoter P_pyrF_ via transcriptomic analysis for biosensor construction. Further investigation suggested that Ihfβ plays a key role in P_pyrF_ activity regulation, while cadaverine and structurally related amines may activate P_pyrF_ through modulation of Ihfβ‐DNA interactions. Stepwise engineering was then performed to enhance biosensor performance, including mutagenesis of the promoter DNA sequence and the Ihfβ sequence, as well as RBS tuning, resulting in more than a 2.33‐fold increase in fold induction. Guided by the biosensor, we implemented a multi‐level engineering strategy that spanned tuning a specific exporter, mining optimized pathways from SCRaMbLE libraries, and genome‐wide identification of beneficial gene targets. These iterative efforts improved cadaverine titer by 44% to a record 114.2 g/L in *Escherichia coli*, concurrently uncovering the critical roles of biofilm formation and carbon flux rewiring for overproduction. Our study establishes a biosensor‐driven framework for optimizing complex pathways and uncovering novel targets in strain engineering.

## Graphical abstract

A high‐throughput screening platform was established by integrating an in vivo cadaverine biosensor with a fluorescence‐activated cell sorting (FACS) system. Biosensor‐driven specific exporter tuning, pathway optimization and genome‐wide target mining were conducted to maximize cadaverine production in *Escherichia coli*. Such genetically encoded biosensors provide powerful tools for accelerating the development of high‐efficient strains for valuable chemical production.

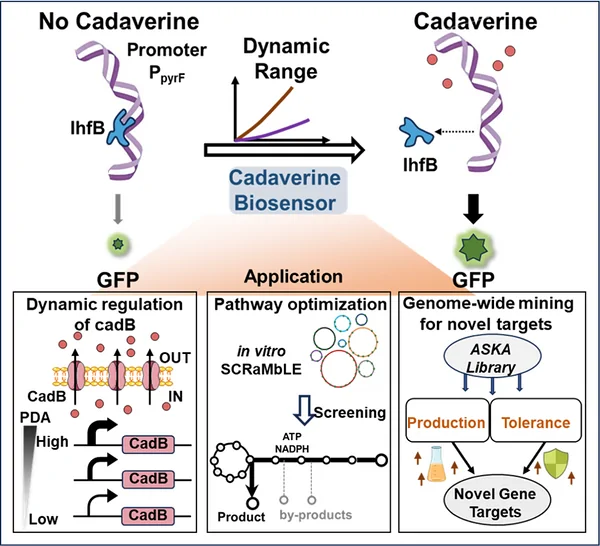

## 1 Introduction

Monomer production from fossil resources has raised significant environmental concerns [1, 2], driving great interest in the development of microbial cell factories for sustainable production of bio‐based alternatives from renewable feedstocks [3, 4]. However, microbial biosynthesis of the target compounds involves complex systemic coordination beyond rational engineering of a few genes closely related to the metabolic network. On the one hand, multiple metabolic genes interact through intricate regulatory networks, and a multi‐modular engineering strategy was often required for efficient metabolic pathway optimization. Additionally, some unrelated metabolic pathways or cellular processes can even unexpectedly impact production as the distal regulatory effects [5, 6]. Therefore, researchers have increasingly adopted high‐throughput genetic tools for systematically generating diverse pathway and genomic variants, promoting the shift of metabolic engineering from “local optimization” to “systematic reconstruction” for the development of high‐performance strains. However, a critical bottleneck still exists where the capacity to generate genetic diversity of strains far exceeds the ability to functionally screen engineered strains as the lack of high‐throughput screening methods for many monomers.

Diamines are critical platform monomers in the synthesis of high‐performance polymers such as polyurethanes and polyamides [7]. Among them, cadaverine has garnered considerable attention as a promising bio‐derived monomer for synthesizing novel high‐performance polymers, such as PA54, PA56, and PDI [8, 9, 10]. For example, compared to petroleum‐based PA66, cadaverine‐derived polyamides exhibit superior properties, including enhanced moisture absorption, biodegradability, and flame retardancy, while maintaining high tensile strength and thermal resistance [11]. Cadaverine biosynthesis has been successfully established in microbial hosts such as *Escherichia coli* [12], mainly through multiple rational engineering strategies including pathway redesign, enzyme optimization, cofactor engineering, and transporter modification. These efforts have led to significant advancements, with a titer of 84.1 g/L reported in *E. coli* [12]. However, further improvement is largely constrained by the reliance on rational, low throughput approaches, as the vast combinatorial space of pathway and genomic variants remains unexplored. Consequently, developing high‐throughput screening (HTS) technologies is crucial for further improve strain performance.

Genetically encoded biosensors have emerged as powerful tools in modern metabolic engineering for screening desired phenotypes or targets by creating libraries of rational or random mutations, which largely accelerate the design‐build‐test cycle for strain development and optimization [13, 14, 15, 16]. Unfortunately, progress in developing diamine‐responsive biosensors was still limited. To date, only a putrescine‐specific biosensor based on the transcription factor PuuR was reported in *E. coli* [17]. This biosensor demonstrates notable selectivity to 1,4‐putrescine, showing minimal cross‐reactivity with structurally similar diamines including 1,3‐diaminopropane, 1,5‐cadaverine, and 1,6‐diaminohexane. Therefore, developing a diamine‐responsive biosensor are highly desirable. Currently, many biosensors are developed based on transcription factors (TFs) that regulate reporter gene expression (e.g., green fluorescent protein, GFP) in response to specific molecular concentrations, enabling detection of target metabolites [18]. More importantly, the growing availability of diverse transcription factor‐promoter pairs has been obtained through genome mining approaches, significantly expanding biosensor design possibilities [19, 20]. These technological advancements motivated our development of novel diamine biosensors, which was subsequently integrated with both pathway and genome‐scale engineering strategies for systematic strain optimization.

Here, we reported the development and application of a genetically encoded biosensor for rapidly pathway optimization and identifying gene targets to improve microbial production of cadaverine. The biosensor was developed by mining the responsive promoter P_pyrF_ based on transcriptome analysis. Further mechanistic analysis revealed that Ihfβ plays a key role in P_pyrF_ activity regulation, while cadaverine and structurally related amines may activate P_pyrF_ through modulation of Ihfβ‐DNA interactions. The fold induction of the biosensor was subsequently optimized by mutating the promoter P_pyrF_ DNA sequence and the IhfB sequence, as well as the RBS tuning. The obtained biosensor was then applied for high‐throughput screening of both (i) pathway libraries generated by in vitro SCRaMbLE‐mediated rearrangements and (ii) the ASKA overexpression collection, uncovering pivotal metabolic genotypes and production‐enhancing targets that were leveraged to construct high‐yield cadaverine producers.

## 2 Results and Discussion

### 2.1 Identifying Cadaverine‐Responsive Promoters in *E. coli*

**FIGURE 1:**
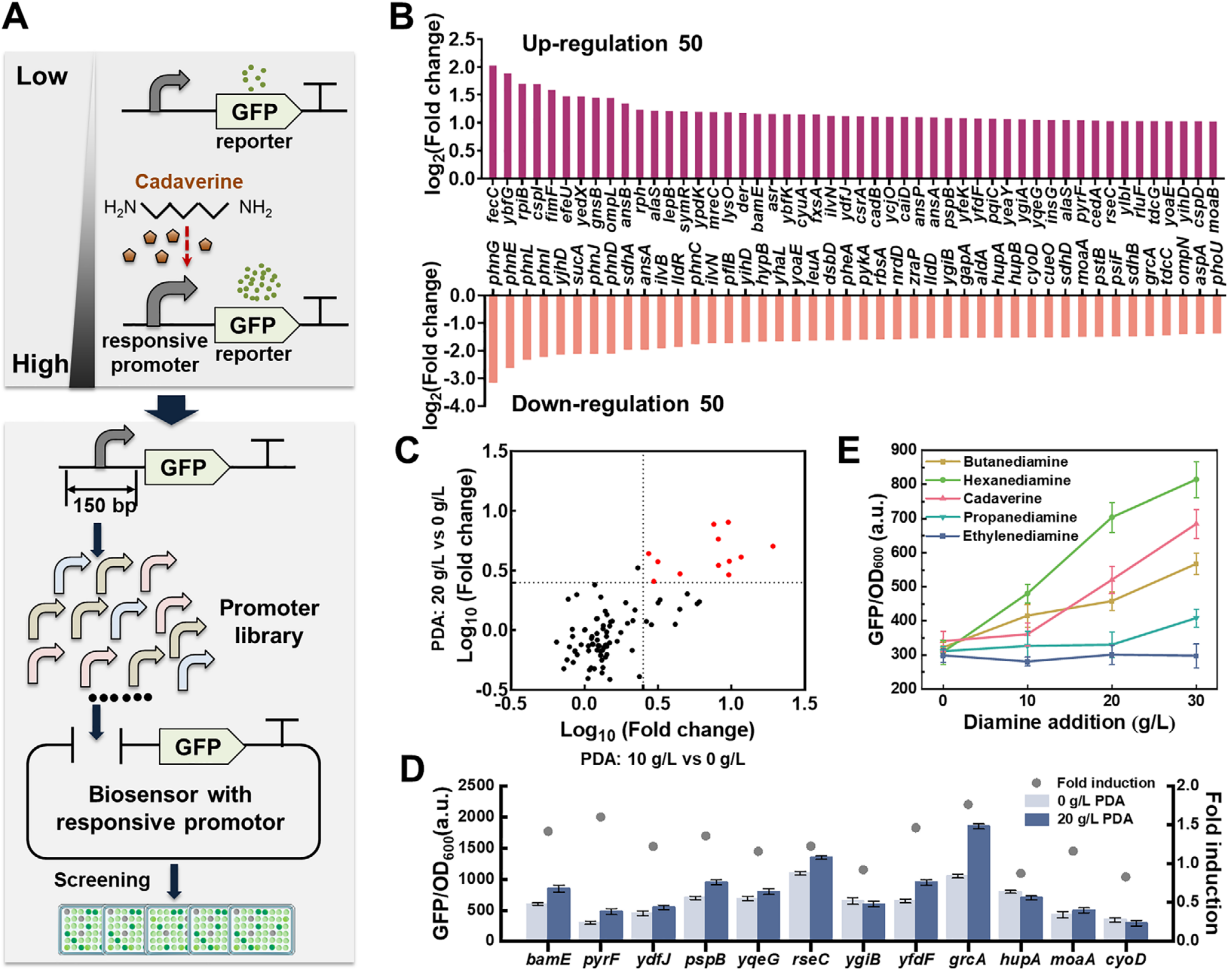
Mining of cadaverine‐responsive promoters based on transcriptome analysis. (A) Schematic diagram of the construction of cadaverine‐responsive promoter library. (B) Comparative transcriptomic analysis of endogenous 100 genes with significant differential expression in response to cadaverine. (C) Fluorescence response profiles of the promoter library to cadaverine exposure. Promoter strength was measured in medium containing 10 or 20 g/L cadaverine and normalized to the control without cadaverine. Red dot: Promoters showing significant upregulation in both 10 and 20 g/L cadaverine. (D) Fluorescence intensity from the 12 candidate cadaverine‐responsive promoters. The fold induction values were calculated as the ratio of the fluorescence intensity at 20 g/L cadaverine to that under uninduced conditions. (E) Fluorescence intensity of the P_pyrF_ promoter to different concentrations of diamines. Fluorescence intensity of GFP normalized to cell density (GFP/OD_600_) was measured at 12 h. GFP/OD_600_ are represented in arbitrary units (a. u.). Data represent mean ± SD of three biological replicates (n = 3). Abbreviation: PDA, cadaverine.

Microorganisms adapt to environmental stress through metabolic reprogramming, typically manifested by differential gene expression [21, 22]. Transcriptomic profiling enables systematic evaluation of these changes, thereby identifying candidate promoters that can be exploited for biosensing or regulatory applications [19, 20]. For example, Yu et al. identified a heme‐responsive promoter via transcriptomics for developing the heme biosensor in *Pichia pastoris* [19]. Here, we previously found that the growth of *E. coli* was markedly suppressed at cadaverine concentrations higher than 20 g/L [23]. Comparative transcriptomics identified 669 significantly differentially expressed genes under high‐concentration cadaverine in *E. coli*. We hypothesized that the native stress response may be exploited to identify cadaverine‐responsive promoter (Figure 1A). Accordingly, the promoters of the top 50 up‐regulated and 50 down‐regulated genes were fused to GFP (Figure 1B, Figure S1), and tested under varying cadaverine concentrations.

To identify cadaverine‐responsive promoters, we initially screened the promoter library under gradient cadaverine concentrations (0–20 g/L). Fluorescence‐based analysis identified 12 candidates that were consistently induced at both 10 g/L and 20 g/L cadaverine (Figure 1C). These candidates were further identified at 20 g/L cadaverine, where P_pyrF_ and P_grcA_ exhibited the highest induction ratios (1.60‐fold and 1.76‐fold, respectively; *p* < 0.01) (Figure 1D). We then characterized P_pyrF_ and P_grcA_ over a finer cadaverine concentration gradient (0–30 g/L). The fluorescence intensity driven by P_pyrF_ increased with increasing cadaverine concentration, whereas fluorescence driven by P_grcA_ decreased (Figure S2). Therefore, P_pyrF_ was selected as the sensing element for subsequent biosensor construction. To evaluate its broader applicability, we further examined the response of P_pyrF_ to four additional diamines (1,2‐ethylenediamine, 1,3‐propanediamine, 1,4‐butanediamine, and 1,6‐hexanediamine). Notably, P_pyrF_ was induced by tested diamines other than 1,2‐ethylenediamine, with fold induction increasing as the carbon chain length increased (Figure 1E).

**FIGURE 2:**
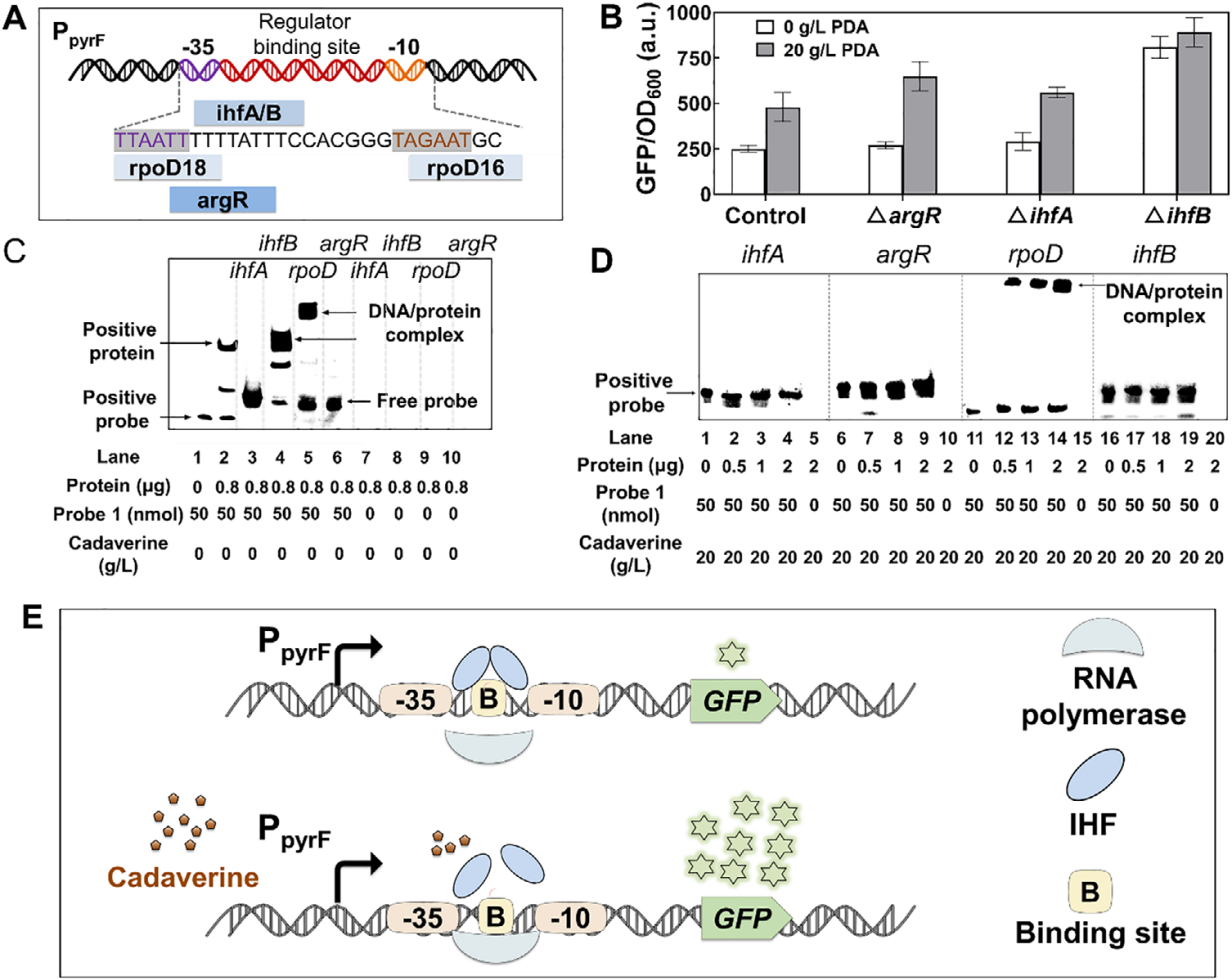
A proposed model for cadaverine‐responsive regulation of the promoter P_pyrF._ (A) Three candidate transcriptional regulators were identified by in silico analysis, including integration host factor (IHF, encoded by *ihfA/ihfB*), arginine repressor (ArgR), and the σ^7^ ^0^ subunit of RNA polymerase (RpoD) with two predicted interaction domains (RpoD16 and RpoD18). (B) The measurement of cadaverine‐response of promoter P_pyrF_ in individual transcription factor deletion mutants (*ΔihfA*, *ΔihfB*, and *ΔargR*) and the strain Control. (C) The binding of transcription factor to promoter DNA without cadaverine addition was characterized based on EMSA, the transcription factors Ihfα and ArgR had no specific DNA binding activity on the probe DNA (lanes 3 and 6), the transcription factors Ihfβ and RpoD had specific DNA binding activity on the probe DNA (lanes 4 and 5), and the controls were shown in lanes 1, 2 and 7–10. Lanes 7–10 were included to confirm that the purified target protein was not contaminated with biotin. Positive probe indicated the biotinylated DNA probe, and positive protein indicated the biotinylated protein control. The DNA‐protein complex appears as shifted bands upon binding of the corresponding protein to the biotinylated DNA probe. (D) The binding of transcription factor to promoter DNA with cadaverine addition was characterized based on EMSA, the transcription factors Ihfα, ArgR, and Ihfβ had no specific DNA binding activity on the probe DNA (lanes 2–4, 7–9, and 17–19), the transcription factor RpoD had specific DNA binding activity on the probe DNA (lanes 12–14), and the controls were shown in lanes 1, 5, 6, 10, 11, 15, 16, and 20. (E) The transcriptional regulatory mechanism of Ihfβ on P_pyrF_.

Biosensors based on transcriptional regulatory factors represent one of the most extensively studied and widely deployed sensing platforms. To rationally engineer a P_pyrF_‐based sensing module, we first investigated the regulatory architecture of the *pyrF* promoter. The upstream region of *pyrF* was analyzed using the BPROM program implemented in the SoftBerry web server [24], revealing a predicted σ^70^ promoter architecture and several potential transcription factor binding motifs. Three candidate regulators were subsequently identified, including Integration Host Factor (IHF, encoded by *ihfA*/*ihfB*), Arginine repressor (ArgR, encoded by *argR*), and RNA polymerase σ^70^ factor (RpoD, encoded by *rpoD*) with two predicted interaction sites (RpoD16 and RpoD18) (Figure 2A). The predicted RpoD16 and RpoD18 motifs overlapped with the −35 and −10 promoter elements, respectively, suggesting that P_pyrF_ is likely recognized by the σ^70^‐dependent transcriptional machinery. To evaluate the potential roles of these transcription factors, we constructed individual deletion mutants (*ΔihfA*, *ΔihfB*, and *ΔargR*) and assessed their effects on P_pyrF_ induction (Figure 2B). Due to the essential role of RpoD in cell viability [25, 26], it was retained in all strains. Deletion of *ihfB* abolished the responsiveness of P_pyrF_ to cadaverine induction, and significantly enhanced basal promoter activity, whereas deletion of *ihfA* or *argR* had little effect (Figure 2B). Consistent with the deletion analysis, *ihfB* overexpression reduced basal P_pyrF_ activity by 51.1% while preserving cadaverine‐dependent induction. In contrast, *ihfA* overexpression reduced the induction fold, and co‐overexpression of *ihfA* and *ihfB* further reduced the fluorescence response (Figure S3). These observations suggested that IhfB may contribute to repression of P_pyrF_, and was important for its diamine‐responsive regulation.

IHF is generally recognized as a heterodimeric DNA‐binding protein composed of IhfA (α) and IhfB (β), which plays important roles in chromosome organization and transcriptional regulation in* E. coli* [27, 28, 29, 30]. Interestingly, despite the canonical role of the Ihfαβ heterodimer, our genetic analyses suggested that Ihfβ is a key determinant for P_pyrF_ regulation. To examine the interaction between these regulators and the P_pyrF_ promoter, we expressed and purified Ihfα, Ihfβ, RpoD and ArgR (Figure S4) for electrophoretic mobility shift assay (EMSA). RpoD specifically bound to the promoter regardless of cadaverine addition (Figure 2D), supporting the prediction that P_pyrF_ is a σ^7^ ^0^‐dependent promoter. In the absence of cadaverine, purified Ihfβ exhibited specific binding to the P_pyrF_ promoter probe, whereas Ihfα alone showed no detectable DNA‐binding activity (Figure 2C). When Ihfα and Ihfβ were mixed, DNA‐protein complexes were also observed (Figure S5). These results indicated that IHF‐containing complexes can interact with the P_pyrF_ promoter in vitro. Upon addition of 20 g/L cadaverine, DNA‐binding signals of both the Ihfβ and the mixed Ihfα/Ihfβ samples were abolished (Figure 2D and Figure S5). Previous studies have shown that both Ihfα and Ihfβ could form homodimers for binding DNA, while the binding affinity of the Ihfβ homodimer is by two orders of magnitude lower than that of the Ihfαβ heterodimer, and Ihfα homodimer is highly unstable [31]. Accordingly, the mixed Ihfα/Ihfβ sample most likely contained multiple IHF assemblies. However, the observation that purified Ihfβ alone binds the P_pyrF_ promoter, together with the genetic evidence that only ihfB deletion abolished cadaverine responsiveness, suggested that Ihfβ might plays a more prominent role in P_pyrF_ regulation. Moreover, MD simulations revealed that the Ihfβ homodimer binds to the P_pyrF_ promoter more stable than the Ihfαβ heterodimer (Figure S6).

Subsequently, we performed molecular dynamics (MD) simulations to evaluate potential the interaction between cadaverine and different IHF species. Cadaverine formed only transient contacts with the Ihfβ homodimer during the initial stage of the simulation and subsequently dissociated, whereas no stable association was observed with the Ihfα homodimer or the Ihfαβ heterodimer (Figure S7). Thus, the MD simulations did not support stable direct binding between cadaverine and IHF. Furthermore, both 1,4‐butanediamine and 1,6‐hexanediamine similarly reduced the DNA‐binding signals of purified Ihfβ and the mixed Ihfα/Ihfβ samples (Figure S8). Thus, the reduction in IHF‐DNA binding was not specific to cadaverine, suggesting that nonspecific physicochemical effects of structurally related amines may contribute to the observed perturbation of the IHF‐DNA interaction, rather than a specific ligand‐protein recognition mechanism.

We next examined whether the response of P_pyrF_ could be explained by the positive charge of the tested compounds. We first examined the response of the P_pyrF_ promoter to three positively charged amino acids, such as lysine, arginine, and histidine, none of which elicited detectable activation (Figure S9A). In contrast, both of the tested polyamines, spermine and spermidine, induced P_pyrF_ promoter activity (Figure S9B). In addition, increasing the extracellular pH to 8 or 9 in the absence of exogenous amines did not further activate P_pyrF_ relative to pH 7 (Figure S9C). Together, these results indicated that the response of P_pyrF_ is not determined by positive charge alone but is also influenced by the molecular properties of the amine compounds. The responsiveness of P_pyrF_ to other amines demonstrated that this sensing system is not strictly specific to cadaverine and can respond to structurally related polyamines. Such cross‐reactivity represents an inherent limitation of the current sensing system, particularly in engineered metabolic backgrounds where cadaverine coexists with other polyamines or amine metabolites. Therefore, potential interference from other responsive amine metabolites should consequently be considered when applying this sensing system across different metabolic contexts.

Based on these genetic, biochemical, and computational analyses, a possible model for P_pyrF_ regulation was proposed (Figure 2E), where Ihfβ is an essential regulatory component required for P_pyrF_ responsiveness. Exposure to cadaverine and structurally related diamines is associated with reduced IHF‐DNA binding and increased promoter activity. However, the physiologically relevant IHF species and the molecular mechanism by which diamines reduce IHF‐DNA binding remain unresolved. In particular, the current results did not establish stable direct ligand binding or a ligand‐specific conformational sensing mechanism. Electrostatic effects may contribute to the observed changes in IHF‐DNA interactions, while the differential responses to structurally distinct amines indicated that the response was not determined by charge alone. Further studies will therefore be required to define the molecular basis of P_pyrF_ activation. Nevertheless, the reproducible dose‐dependent response of P_pyrF_ to cadaverine and related diamines establishes it as a useful diamine‐responsive transcriptional element for biosensor engineering and dynamic regulation.

### 2.2 Mutation Optimization of Cadaverine‐Responsive Promoter P_pyrF_

**FIGURE 3:**
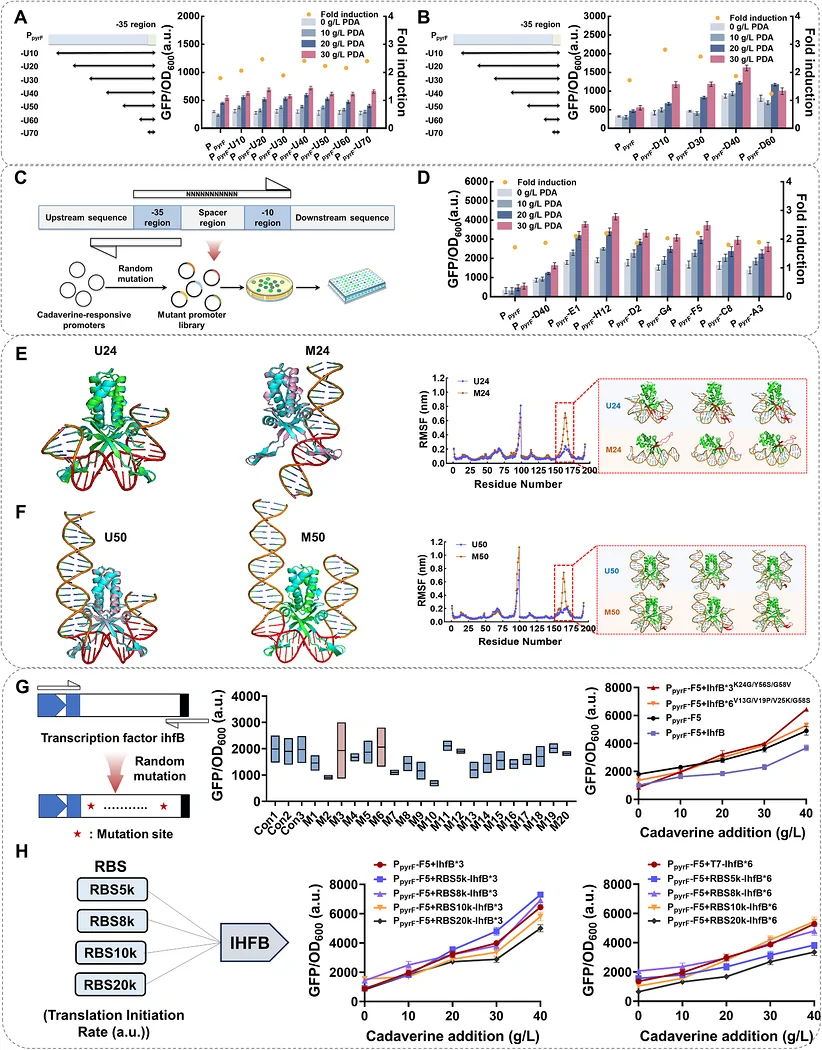
Truncation and random mutagenesis to increase the properties of cadaverine‐sensing biosensor. (A) Identification of the suitable P_pyrF_ regulatory region by truncating upstream of the promoter. Serial 10‐bp deletions within the upstream region (−65 to −1 relative to the −35 hexamer) generated seven mutants (P_pyrF_‐U10‐GFP, P_pyrF_‐U20‐GFP, P_pyrF_‐U30‐GFP, P_pyrF_‐U40‐GFP, P_pyrF_‐U50‐GFP, P_pyrF_‐U60‐GFP, P_pyrF_‐U70‐GFP). (B) Identification of the suitable P_pyrF_ regulatory region by truncating downstream of the promoter. Serial 10‐bp deletions within the downstream region (+1 to +58 relative to the −10 hexamer) generated four mutants (P_pyrF_‐D10‐GFP, P_pyrF_‐D30‐GFP, P_pyrF_‐D40‐GFP, P_pyrF_‐D60‐GFP). (C) Schematic diagram of random mutagenesis of the spacer region in promoter P_pyrF_‐D40. (D) Response plot of beneficial mutants (P_pyrF_‐GFP, P_pyrF_‐D40‐GFP, P_pyrF_‐E1‐GFP, P_pyrF_‐F5‐GFP, P_pyrF_‐H12‐GFP, P_pyrF_‐C8‐GFP, P_pyrF_‐D2‐GFP, P_pyrF_‐A3‐GFP, P_pyrF_‐G4‐GFP) in graded concentrations of cadaverine (0‐30 g/L). The fold induction values were calculated as the ratio of the fluorescence intensity at 30 g/L cadaverine to that under uninduced conditions. (E) Ihfαβ‐unmutated Promoter (24‐base pair) (Left) and Ihfαβ‐mutated Promoter (24‐base pair) (Right) composite structure predicted by AlphaFold3. The DNA binding site of Ihfαβ is colored in Red and the structure of the predicted Ihfαβ heterodimer is aligned with the experimental structure from PDB 1IHF (Blue). Comparison of RMSF between the Ihfαβ‐M24 (24‐bp mutated Promoter) complex and the Ihfαβ‐U24 (24‐bp Unmutated Promoter) complex. (F) Ihfαβ‐unmutated Promoter (50‐base pair) (Left) and Ihfαβ‐mutated Promoter (50‐base pair) (Right) composite structure predicted by AlphaFold3. The red DNA sequences represent the binding sites of IHF on the mutated and unmutated promoters, respectively. Comparison of RMSF Between Ihfαβ‐M50 (50‐bp mutated Promoter) complex and the Ihfαβ‐U50 (50‐bp unmutated promoter) complex. (G) Schematic diagram of random mutagenesis of transcription factor IhfB. Screening of mutant transcription factor ihfB library using biosensor P_pyrF_‐F5 under stress of 20 g/L cadaverine. Response plot of P_pyrF_‐F5 in the beneficial transcription factor IhfB randomly mutaion strain under graded concentrations of cadaverine (0‐40 g/L). (H) Optimization of the RBS strength for the mutant transcription factor IhfB*3 and IhfB*6. Fluorescence intensity of GFP normalized to cell density (GFP/OD_600_) was measured at 12 h. Data represent the mean ± SD of three biological replicates.

To enhance biosensor performance, strategies like optimizing promoter sequence or mutating transcription factors are commonly employed [32]. In *E. coli*, the regulation mechanism of P_pyrF_ remains uncharacterized. We therefore employed two optimization strategies, including systematic truncation analysis of promoter elements, and random mutagenesis of the spacer region. In the wild‐type promoter P_pyrF_, the 150‐bp region upstream of P_PyrF_ was fused with GFP. We first divided the 150‐bp sequence into upstream (−65 to −1 relative to the −35 hexamer) and downstream (+1 to +58 relative to the −10 hexamer) segments. Serial 10‐bp deletions within the upstream region generated seven mutants, and no significant change in fluorescence intensity was observed (Figure 3A). In contrast, progressive deletion downstream of the −10 hexamer identified P_pyrF_‐D40, a 40‐bp truncation that increased the overall fluorescence intensity the most and raised the fold induction to 1.87‐fold (p < 0.01) (Figure 3B). This variant was selected for further optimization.

We then generated a mutation library by randomizing the 15‐bp spacer sequence in P_pyrF_‐D40 (Figure 3C). Their response to cadaverine was then screened via fluorescence assays (Figure S10). Seven mutants exhibited significantly improved fluorescence value increasing from 320 to a maximum of 1,880, among which P_pyrF_‐F5 demonstrated a 5.5‐fold increase in baseline fluorescence while maintaining a highest induction fold (2.19‐fold, *p* < 0.01) (Figure 3D). Sequencing revealed that its spacer GC content increased from 40% to 80% (Figure S11).

We further employed structural modeling to elucidate IHF‐promoter interactions. The structure of the Ihfαβ heterodimer was predicted with AlphaFold3 (Figure S12), and an optimized DNA helices models was generated via the nucleic‐acid modeling tool 3DNA (Figure S13). The results revealed that IHF bound the P_pyrF_ promoter in a U‐turn configuration, bending DNA by over 160° (Figure 3E). This bend reverses the direction of the helix within a very short span and is stabilized by kinks where proline residues from IHF intercalate between bases. In addition, structural analysis revealed that IHF's binding interface features β‐ribbon “arms” that insert into the minor groove of DNA at the bend, providing direct structural evidence for Ihfβ’s regulatory role as a transcriptional regulator of P_pyrF_.

As the increased performance by mutant P_pyrF_‐F5, we further investigated the alterations in binding affinity between IHF and its binding sites following mutation. Structural predictions showed that IHF exhibits reduced binding capability with the mutant P_pyrF_ (Figure 3E), and only a single β‐ribbon “arms” successfully engaged with and inserted into the DNA minor groove. FoldX calculations also confirmed significantly weakened binding affinity, from −13.8 kcal/mol for wild‐type P_pyrF_ to 0.26 kcal/mol for the mutant P_pyrF_‐F5. This pronounced difference indicates that specific mutations in the promoter disrupt IHF‐DNA interaction, likely by altering key nucleotide positions critical for structural recognition. MD simulation further supported these observations. In the mutant complex (M24), residues in the positions of 150–175 exhibited significantly elevated RMSF values compared to the wild‐type complex U24 (Figure 3E), indicating enhanced flexibility and structural instability. Structural visualizations further confirmed high conformational flexibility and weakened protein‐DNA interactions within this region for M24 (Figure 3E). Conversely, the corresponding region in U24 displayed relatively lower RMSF values (Figure 3E), consistent with a structurally stable complex. These observations suggested that the mutation of DNA binding site disrupted the recognition of β‐ribbon “arms” to promoter, seriously affecting the structural stability of the complex.

In addition, computational analysis revealed persistent, albeit weak, interaction at the mutated promoter regions. We subsequently performed detailed characterization of this residual binding activity by using a 50‐base pair promoter DNA sequence. AlphaFold3 simulations indicated that IHF binds well to both mutated and unmutated promoters with distinct binding motifs: 5’‐AAATAAAAAATTAA‐3’ for the unmutated promoter and 5’‐ATCGCAACTGTTAA‐3’ for the mutated one (Figure 3F), consistent with predicted IHF recognition motifs (Table S1). Subsequent MD analysis revealed that the two β‐ribbon arms of IHF embedded stably into the wild‐type promoter, with a phenylalanine residue deeply inserting into the DNA minor groove (Figure 3F). In the mutant promoters, however, the arms were loosely associated, exhibiting pronounced fluctuation and reduced shape complementarity (Figure 3F). The decrease in the binding affinity of the IHF‐DNA complex explained the phenomenon of increased fluorescence intensity after promoter mutation (Figure 3D).

To further enhance the performance of P_pyrF_‑F5, we performed random mutagenesis on the transcription factor IhfB. Using the biosensor P_pyrF_‑F5‑GFP, we screened a random mutagenesis library of IhfB in the presence of 20 g/L cadaverine and identified two mutants with improved fold induction: IhfB*3 (K24G, Y56S, G58V) and IhfB*6 (V13G, V19P, V25K, G58S) (Figure 3G). Both mutants were individually validated using the P_pyrF_‐F5‐GFP biosensor in media containing gradient concentrations of cadaverine. The results showed that IhfB*3 and IhfB*6 could increase the induction fold (30 g/L vs. 0 g/L) of the biosensor to 4.62‐fold (*p* < 0.01) and 2.86‐fold (*p* < 0.01), respectively (Figure 3G). Subsequently, the ribosomal binding site (RBS) strength of these two mutants was optimized, with the corresponding RBS sequences listed in Table S2. As shown in Figure 3H, optimization of the RBS strength of IhfB*3 to 5k further enhanced the induction fold (30 g/L vs. 0 g/L) of the biosensor P_pyrF_‑F5‑GFP to 5.33, representing a 2.33‑fold improvement compared to the control P_pyrF_‑GFP. In contrast, similar optimization of IhfB*6 did not significantly improve the fold induction of the biosensor P_pyrF_‑F5‑GFP (Figure 3H). Based on these studies, we developed an efficient cadaverine response biosensor with potential for high‐throughput screening.

### 2.3 Design of a Biosensor‐Based Screening Platform for the Rapid Optimization of Multigene Pathway to Enhance Cadaverine Biosynthesis

**FIGURE 4:**
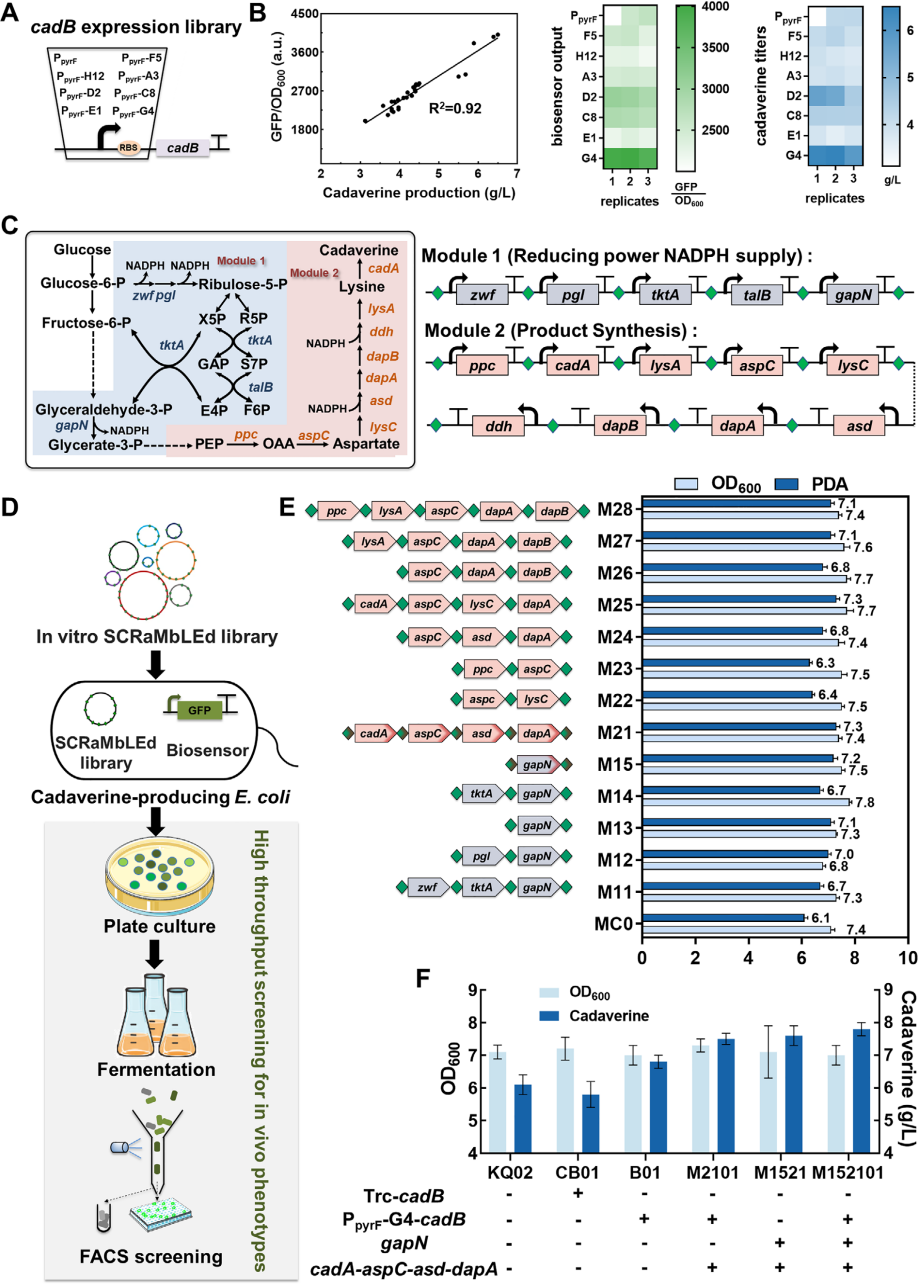
Screen of *cadB* expression library and SCRaMbLEd strains for high cadaverine production using cadaverine biosensor. (A) The expression regulation of the gene *cadB* by different cadaverine‐responsive promoters. (B) Correlation of cadaverine production with biosensor fluorescence outputs for the screened microbial strains. A straight‐line curve was fitted using GraphPad Prism linear regression fit, R^2^  =  0.92. (C) Diagram of the metabolic pathway from glucose to cadaverine. Modular design and cluster assembly of cadaverine metabolic pathway on plasmid BAC. (D) Schematic diagram of high‐throughput screening and genotypic analysis of in vitro SCRaMbLEd library. (E) The screened strains were verified by shaking flask fermentation and tested for genotype. (F) Verification of cadaverine production and OD_600_ of the high‐performing cadaverine producing strains. Three biological replicates; Error bars represent standard deviations. Abbreviations: P, phosphate; *zwf*, glucose‐6‐phosphate‐1‐dehydrogenase; *cadA*, lysine decarboxylase; *lysA*, diaminopimelate decarboxylase; *ppc*, phosphoenolpyruvate carboxylase; *aspC*, aspartate aminotransferase; *lysC*, aspartokinase III; *asd*, aspartate semialdehyde dehydrogenase; *dapA*, dihydrodipicolinate synthetase; *dapB*, dihydrodipicolinate reductase; *ddh*, diaminopimelate dehydrogenase from *Corynebacterium glutamicum*; *tktA*: transketolase I; *talB*: transaldolase.

To further evaluate the applicability of the cadaverine biosensor in actual fermentation conditions, we first constructed a panel of engineered strains exhibiting different cadaverine production levels by dynamically regulating the expression of the cadaverine transporter gene *cadB* using P_pyrF_ and its seven mutant promoters (Figure 4A). CadB is a transmembrane cadaverine/lysine antiporter that mediates both efflux and uptake of cadaverine depending on physiological conditions [33]. Overexpression of *cadB* in *E. coli* has been previously reported capable of improving cadaverine production [34]. Using these strains, we examined both the GFP fluorescence and cadaverine fermentation titer. A positive correlation was observed between GFP fluorescence and cadaverine production (Figure 4B), demonstrating that the output of the biosensor could be used as a readout of the production state. Intracellular cadaverine showed a consistent increasing trend with increasing production (Figure S14). Notably, a reduced detection threshold of the biosensor was observed under endogenous synthesis conditions. Similar phenomenon has been also reported in several previous studies, which were presumably associated with intracellular effector‐promoter interactions facilitated by the alleviation of membrane transport limitations [35, 36, 37]. As P_pyrF_‐G4‐driven *cadB* expression resulted in the highest cadaverine production among the tested promoter variants (Figure 4B), we further examined its dynamic behavior during fermentation. The results showed that the *cadB* transcriptional level was increased over the course of fermentation (Figure S15A). Importantly, intracellular cadaverine did not decrease throughout fermentation (Figure S15B), and the biosensor signal remained consistent with the intracellular cadaverine levels, with no apparent progressive attenuation following *cadB* expression (Figure S15C). Based on the above biosensor‐guided identification of P_pyrF_‐G4‐driven *cadB* expression as a target, we introduced this engineering strategy into the KQ02 control strain. The resulting strain B01 produced 6.8 g/L cadaverine in shake‐flask fermentation, representing a 12% increase over KQ02 (Figure 4F). To determine whether the production benefit was associated with dynamic regulation of *cadB* expression, we additionally constructed a strain CB01 expressing *cadB* constitutively under the Trc promoter, which showed a comparable strength to induced P_pyrF_‐G4 (Figure S16). Under the same conditions, the constitutive‐expression strain CB01 produced 5.8 g/L cadaverine, lower than B01. These results supported the effectiveness of P_pyrF_‐G4‐driven dynamic *cadB* expression and also demonstrated that the biosensor‐based screening strategy could be employed for identifying high‐performing strains.

Efficient synthesis of products typically involves a variety of biological processes like precursor synthesis and cofactor supply. Optimizing these metabolic pathways is therefore critical for developing efficient microbial cell factories. SCRaMbLE system has emerged as a promising tool to generate diverse DNA structural variations [38]. Specifically, Wu et al. developed an in vitro SCRaMbLE system enabling rapid pathway optimization by rearranging relevant transcription units and provides a direct correlation between phenotype and pathway genotype [38]. Here, we applied this strategy to construct a pathway library for cadaverine biosynthesis, followed by biosensor‐based screening to rapidly optimize the pathway. First, the pathway was divided into two modules: the NADPH supply module (Module 1) and the synthesis module from phosphoenolpyruvate (Module 2) (Figure 4C). Module 1 contained 5 genes (*zwf*, *pgl*, *tktA*, *talB*, and *gapN*), and Module 2 contained 9 genes (*ppc*, *cadA*, *lysA*, *aspC*, *lysC*, *asd*, *dapA*, *dapB*, and *ddh*). The genes of each module were clustered and assembled into a bacterial artificial chromosome (BAC) with trc promoters and flanking loxPsym sites (Figure 4C). Subsequently, the two modules were rearranged in vitro using Cre recombinase to generate a SCRaMbLEd pathway library. Gel electrophoresis of the linearized library revealed diverse DNA bands, indicating high efficiency and diversity of the SCRaMbLEd pathway library (Figure S17A). The library was then transformed into NEB 10‐beta, yielding numerous variant strains. Sequencing of randomly selected clones also confirmed the occurrence of rearrangement events in both modules (Figure S17B,C). At the meantime, the two SCRaMbLEd pathway libraries were transformed into a lysine producing strain KQ01 harboring cadaverine biosensor and lysine decarboxylase, yielding SCRaMbLEd strain libraries of M1 and M2 (Figure 4D). Both the M1 and M2 libraries exhibited higher fluorescence intensity and cadaverine production than the control strain MC (Figure S18), indicating the presence of beneficial mutations within the libraries. Following this, we performed fluorescence‐activated cell sorting (FACS) on the fermentation broth of two SCRaMbLEd library (Figure 4D), successfully isolating single cells with high fluorescence intensity (Figure S19). Through screening, five improved strains were obtained from Module 1 SCRaMbLEd strains and eight from Module 2, all of which exhibited significantly higher fluorescence than the control (Figure S20A). Strains with differential fluorescence intensity during the screening process were selected for high‐performance liquid chromatography (HPLC) analysis, and a positive correlation was observed between fluorescence intensity and cadaverine production (Figure S20B). To accurately evaluate production performance without biosensor interference, the sensor plasmid was removed from the 13 top SCRaMbLEd variants. Shake‐flask fermentation demonstrated that all variants outperformed the control strain MC0 in both biomass accumulation and cadaverine production (Figure 4E). Notably, strains M15 and M21 achieved the highest titers of 7.2 and 7.3 g/L, respectively, substantially exceeding the control (6.1 g/L). Further sequencing of these 13 high‐performing variants revealed substantial diversity in their pathway genotypes, confirming the effectiveness of the SCRaMbLE strategy in generating diverse pathway gene combinations. Importantly, these genetic rearrangements indeed conferred the enhanced production phenotypes. The rearranged Module 1 variant M15 retained only the* gapN* gene, which encodes a glyceraldehyde 3‐phosphate dehydrogenase from *Streptococcus mutans*. This enzyme converts NAD^+^ to NADP^+^, and has been employed as an engineering strategy to optimize NADPH supply in cells [39]. This finding suggested that simplifying the module to enhance the NADPH regeneration flux is a more effective strategy. For the strain M21 containing rearranged module 2 variant, four genes *cadA, aspC, asd*, and *dapA* were retained, further indicating that reinforcing lysine decarboxylase and upstream precursor synthesis is crucial for directing carbon flux toward cadaverine biosynthesis.

To systematically evaluate the combinatorial effect of these beneficial genotypes, we constructed a series of engineered strains. First, integrating the expression of *cadA‐aspC‐asd‐dapA* with CadB yielded strain M2101, which achieved 7.5 g/L cadaverine with a yield of 0.37 g/g _glucose_. We then co‐expressed the optimal pathway genotypes of *cadA‐aspC‐asd‐dapA* and *gapN* to generate strain M1521, which further increased the titer to 7.6 g/L. On this basis, further expression of *cadB* was conducted to generate strain M152101, achieving a cadaverine production of 7.8 g/L with a yield of 0.39 g/g _glucose_, representing a 28% increase over the control strain (Figure 4F). Among the genotypes associated with the high‐yielding cadaverine producers, alleles of *aspC*, *dapA* and *gapN* were recovered at the highest frequencies, providing clear guidance for subsequent strain engineering. These results demonstrated that our biosensor‐driven screening platform is highly effective for rapidly identifying high‐performance strains from complex DNA libraries. The platform successfully established a direct link between genotype and production phenotype, allowing us to decipher non‐intuitive yet beneficial genetic configurations. The stepwise modular optimization, guided by the screening outcomes, ultimately led to a significant yield improvement, validating the entire strategy for optimizing complex multigene pathways.

### 2.4 Using the Biosensor to Identify Gene Targets Beneficial for Cadaverine Overproduction

**FIGURE 5:**
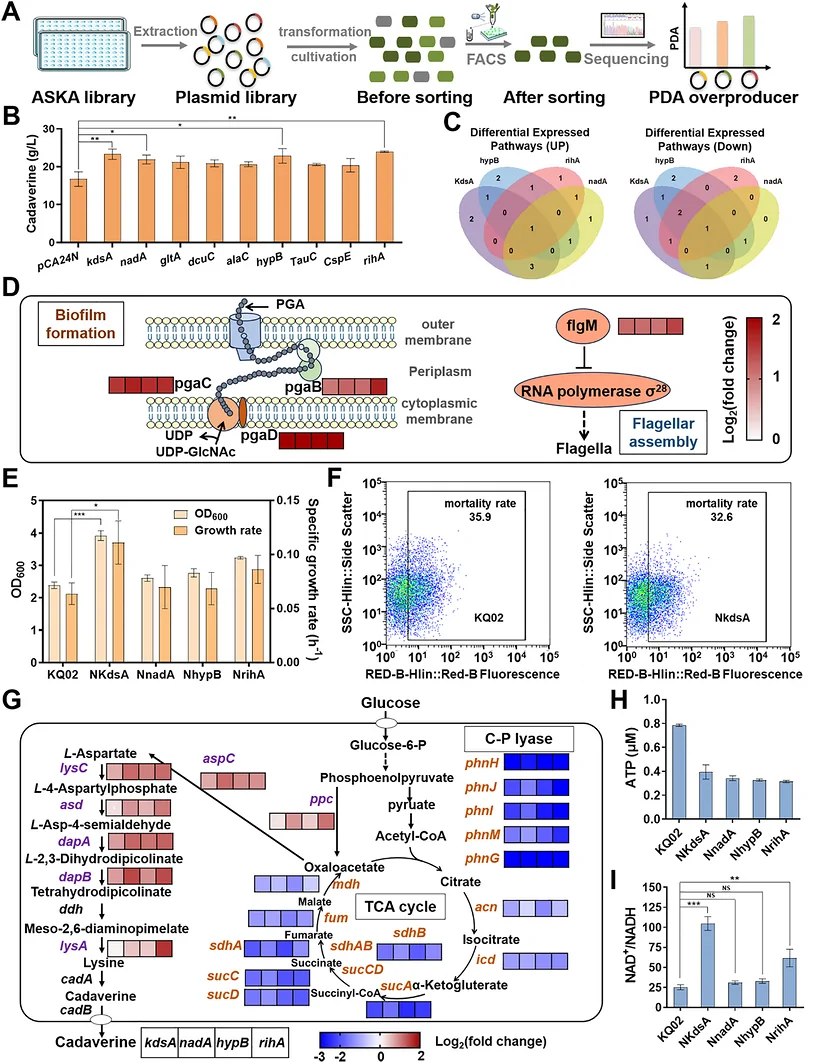
Biosensor‐guided genome‐scale overexpression library screening identifies novel gene targets for enhanced cadaverine production and elucidates their mechanisms. (A) Schematic illustration of genome‐scale overexpression library screening to identify gene targets conferring cadaverine overproduction based on our developed cadaverine biosensor. (B) Cadaverine production by engineered strains overexpressing the candidate gene targets. (C) Venn diagram of differentially expressed genes across the engineered strains NkdsA, NhypB, NrihA, and NnadA vs. the control. (D) Impact of the four gene targets on the transcriptional levels of biofilm formation‐related genes. (E) Effects of the four gene targets on strain tolerance to cadaverine (characterization of biomass under 30 g/L cadaverine). (F) Measurement of the mortality rate of strains KQ02 and NkdsA at mid fermentation stage (16 h). (G) Impact of the four gene targets on the transcriptional levels of genes associated with the TCA cycle and precursor synthesis. (H) Intracellular ATP metabolic levels in strains overexpressing related gene targets. (I) Intracellular redox levels in strains overexpressing related gene targets; Three biological replicates; Error bars represent standard deviations. *p < 0.05, **p < 0.01, ***p < 0.001, and NS indicates not significant as determined by t‐test.

As the intricate interconnectivity within cellular networks [40], it remains challenging to predict beneficial genes that can actually enhance the synthesis of target products [41]. Genome‐wide identification strategies have been successfully applied to uncover gene targets for enhancing chemical production (e.g., fatty acids), or stress tolerance like desiccation [6, 42]. To identify gene targets responsible for enhancing cadaverine production, we performed a genome‐scale overexpression library screen combining with the biosensor and FACS (Figure 5A). The library was constructed by co‐transforming ASKA library plasmids, together with the biosensor plasmid P_pyrF_‐F5‐GFP‐*cadA* into strain KQ01. A control strain harboring the empty vector pCA24N along with the biosensor plasmid was included for baseline comparison. Following induction and cultivation, cells with elevated fluorescence were isolated, and overexpressed genes in selected clones were subsequently identified by sequencing and BLAST analysis, allowing for uncovering candidate genes that potentially contributed to enhanced cadaverine production. As a result, the top 9 candidate genes with significantly higher cadaverine production were selected (Figure S21). The control strain produced 16.75 g/L cadaverine. In contrast, 9 engineered strains, each overexpressing a gene target of *kdsA*, *nadA*, *gltA*, *dcuC*, *alaC*, *hypB*, *TauC*, *CspE* or *rihA*, showed enhanced cadaverine production (Figure 5B). Four targets (*kdsA*, *hypB*, *rihA*, *nadA*) led to particularly significant improvement, yielding 23.31, 22.84, 23.96, and 21.93 g/L cadaverine, respectively (Figure 5B). These values correspond to 1.39, 1.36, 1.43, and 1.31‐fold increases over the control strain. Taken together, our genome‐scale overexpression screening successfully identified several novel gene targets for cadaverine overproduction.

Most of these newly identified beneficial genes are implicated in critical cellular processes, including maintenance of redox homeostasis, transcriptional regulation, biofilm formation, and other physiological processes. Among them, *kdsA* encodes 3‐deoxy‐*_D_*‐manno‐octulosonic acid 8‐phosphate synthase, a key enzyme involved in lipopolysaccharides (LPS) biosynthesis [43, 44, 45]. Engineering of kdsA has been shown to compromise outer membrane integrity and disrupt cell division, ultimately resulting in impaired bacterial lysis and death [44]. The *nadA* gene encodes quinolinate synthase, essential for NAD biosynthesis, a central coenzyme in microbial redox reactions [46]. The *hypB* gene encodes a GTPase participating in hydrogenase maturation and the nickel ion transport and integration, which has been reported to enhance acid resistance in *E. coli* [47, 48, 49, 50]. The *rihA* gene encodes a pyrimidine‐specific ribonucleoside hydrolase that can directly hydrolyze cytidine into cytosine and ribose [51, 52], which is crucial for DNA replication, transcription, and translation. To elucidate the metabolic influence of these four genes during the cadaverine production, we constructed strains overexpressing each gene targets (*kdsA*, *hypB*, *rihA*, and *nadA*) for fed‐batch fermentation, and performed transcriptomic analysis on samples taken at mid fermentation stage (Figure S22). Comparative analysis revealed that all four engineered strains exhibited significantly transcriptional changes relative to the control strain KQ02 (Figure S23).

Further KEGG pathway enrichment analysis and Venn diagram highlighted biofilm formation as a common upregulated pathway across all four engineered strains (Figure 5C and Figure S24). In detail, four genes of *flgM, pgaB, pgaC, and pgaD* associated with biofilm formation were markedly up‐regulated in the four engineered strains, exhibiting 2.1‐ to 4‐fold increase compared to the control (Figure 5D). The *flgM* gene is an anti‐sigma factor responsible for the regulation of flagellar assembly [53], and *pgaBCD* encode enzymes involved in poly‐β‐1,6‐N‐acetyl‐*_D_*‐glucosamine (PGA) synthesis, a key component of the biofilm matrix [54]. The previous study has demonstrated that biofilm formation represents a key bacterial adaptive strategy to cope with environmental stress [55]. During cadaverine fermentation, product accumulation can cause cell membrane damage [34]. The increased gene expression in biofilm formation may enhance the organism's tolerance to the product cadaverine by forming a protective matrix, maintaining cellular stability, and thus supporting long‐term fermentation and higher cadaverine production. To verify this, we performed crystal violet staining under fermentation conditions. At 36 h, the NkdsA and NhypB strains showed increased biofilm formation relative to the control; by 48 h, all four engineered strains exhibited significantly enhanced biofilm formation, supporting the contribution of biofilm to the improved fermentation performance (Figure S25). To assess whether the gene targets of *kdsA*, *hypB*, *rihA*, and *nadA* could affect strain tolerance to cadaverine, we cultivated these strains with an initial 30 g/L cadaverine, a concentration previously shown to markedly inhibit growth [23]. As we expected, all engineered strains exhibited improved tolerance, achieving higher final biomass and growth rate (Figure 5E). Notably, the strain overexpressing *kdsA*, a gene directly implicated in the synthesis of the biofilm key component LPS, showed the most pronounced enhancement, with biomass and maximum specific growth rate increasing by approximately 1.6‐fold and 1.7‐fold, respectively (Figure 5E). Taking *kdsA* as an example, we further conducted bacterial SYTO/PI staining experiments to characterize cell membrane stability during the cadaverine production. The results showed that the mortality rate in the control strain KQ02 (35.9%) was higher than that in the NkdsA strain (32.6%) (Figure 5F). Together, these results suggested a correlation between biofilm formation and improved production across the four strains. Further genetic validation, for example through targeted manipulation of *pgaC*/*pgaD* or other biofilm‐associated genes to regulate cadaverine production could be explored in future studies.

Conversely, analysis of downregulated pathways identified the tricarboxylic acid (TCA) cycle as a commonly suppressed pathway across all strains (Figure 5C and Figure S24). As the central pathway for carbon and energy metabolism, the down‐regulation of TCA cycle suggested that these four gene targets could affect central carbon flux or induce an energy perturbation. Detailed transcriptomic analysis revealed that most genes in the TCA cycle were significantly repressed, whereas genes involved in the precursor lysine biosynthesis were markedly up‐regulated (Figure 5G). These findings indicated that the four target genes could redirect the carbon flux from the TCA cycle toward product synthesis, thereby enhancing cadaverine production. In terms of energy metabolism, all engineered strains exhibited reduced intracellular ATP levels compared to the control (Figure 5H). At the meantime, the downregulation of oxidative phosphorylation genes (e.g., *phnH*, *phnJ*, *phnI*, *phnM*, and *phnG*) were also observed (Figure 5G). In addition, measurements of intracellular NAD^+^ and NADH revealed that the four genes altered the NAD^+^/NADH ratio (Figure 5I and Figure S26). These results demonstrated that these genes also rewired intracellular redox and energy metabolism with reprogramming carbon flux. Together, these genes enabled synergistic strain improvement through dual‐track regulation of “enhanced tolerance” and “metabolic network optimization”.

### 2.5 Combinatorial Optimization of Beneficial Gene Targets for High‐Yield Production of Cadaverine in *E. coli*

**FIGURE 6:**
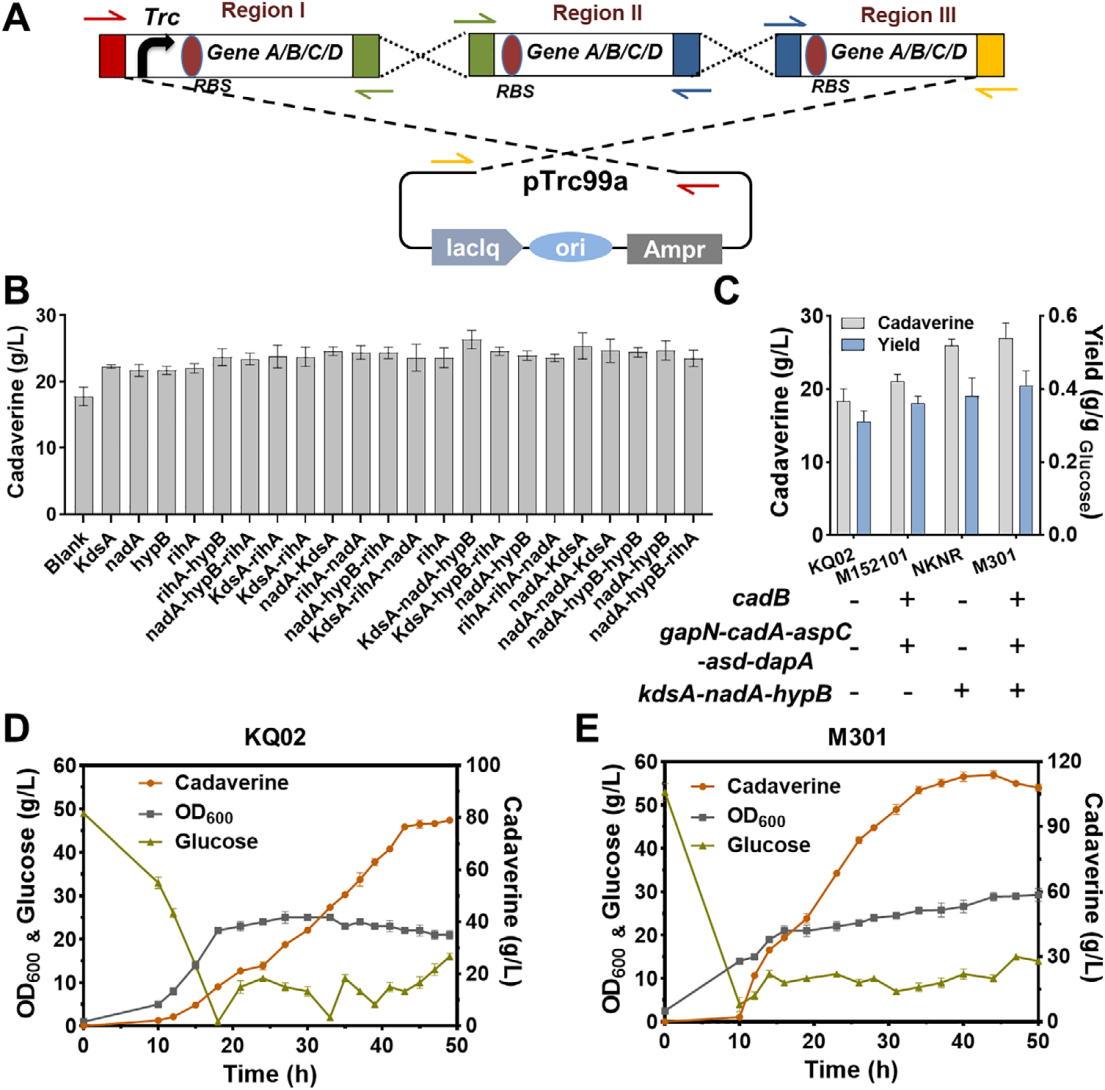
Combination optimization of high‐yield cadaverine targets and fed‐batch fermentation in a 5L Fermenter. (A) Construction process of multigene combinatorial library. A: *kdsA*; B: *nadA*; C: *rihA*; D: *hypB*. (B) Rescreening of the multi‐gene library in shake flask fermentation and sequencing. (C) Verification of cadaverine production and yield of the high‐performing cadaverine producing strains. (D,E) Fed‐batch fermentation of the strains KQ02 and M301 in a 5 L bioreactor. Three biological replicates; Error bars represent standard deviations.

Based on the above identification of four beneficial genes (*kdsA*, *nadA*, *hypB*, and* rihA*) (Figure 5B), we further implemented a systems‐level strategy of combinatorial multiplex perturbation of these beneficial genes to enhance cadaverine production. A combinatorial library was constructed by randomly assembling these four genes via a standardized assembly strategy (Figure 6A). Analysis of the positive clones revealed that three‐fragment assemblies accounted for 81.8%, with two‐fragment and single‐fragment assemblies accounting for 9.1% each (Figure S27). The library was then subjected to high‐throughput screening using our developed biosensor, and 37 clones with elevated fluorescence was initially identified in 96‐well plates. Subsequent fed‐batch shake‐flask fermentation rescreening yielded 18 strains outperforming the control, and the genotypes of these selected strains were confirmed by sequencing (Figure 6B). The best‐performing strain, designated NKNR, harbored the *kdsA‐nadA‐hypB* combination. This strain achieved a cadaverine titer of 26.4 g/L with a yield of 0.38 g/g _Glucose_, representing a 23% increase in yield over the control strain KQ02.

Building on earlier pathway optimizations, including dynamic regulation of *cadB* using promoter P_pyrF_‐G4, as well as enhanced expression of genes *cadA*, *aspC*, *asd*, *dapA*, and *gapN*, could effectively improve cadaverine production. To systematically integrate these optimization strategies, we further integrated these elements with the *kdsA*‐*nadA*‐*hypB* combination into the genome, generating the final engineered strain M301. In shake flask fermentation, M301 produced 27 g/L cadaverine with a yield of 0.41 g/g _glucose_, a 32% increase in yield compared to the control strain (Figure 6C). To further evaluate its production activity, we performed a fed‐batch fermentation in a 5‐L fermenter. The strain M301 produced 114.2 g/L of cadaverine at a yield of 0.37 g/g _glucose_ and a productivity of 2.59 g/L/h (Figure 6D). This marked a 44% improvement in titer, a 23% higher yield, and a 62% increase in productivity compared to the control strain (79 g/L, 0.30 g/g _glucose_, 1.60 g/L/h) (Figure 6D). Notably, the biomass of M301 continued to increase throughout the 0–44 h, unlike the control, which showed decline likely due to the cadaverine accumulation. To our knowledge, this study reports the highest cadaverine titer in *E. coli* and the highest cadaverine productivity achieved to date through microbial fermentation.

## 3 Conclusion

We developed a cadaverine biosensor based on a cadaverine‐responsive promoter P_pyrF_ and also proposed a possible Ihfβ‐involved regulatory model. Subsequently, through mutagenesis of the promoter DNA sequence and the Ihfβ sequence, as well as RBS tuning, the fold induction of the biosensor can be increased by more than 2.33‐fold. Using this biosensor, we established an efficient screening platform combined with FACS. Its high reliability was demonstrated by a strong positive correlation (R^2^ = 0.92) between fluorescence intensity and cadaverine titer. Moreover, this biosensor exhibited an adaptability for monitoring other diamines, thereby offering a robust and versatile tool for diamine biosynthesis. Consequently, this biosensor was further applied to guide the multi‐level engineering of *E. coli* for enhanced cadaverine production, from tuning a defined functional gene, to mining optimized pathways from SCRaMbLE libraries, and to genome‐wide identifying novel targets. We engineered an *E. coli* strain that achieved the highest cadaverine titer (114.2 g/L) and the highest productivity (2.59 g/L/h) reported in this host, with a 62% increase in productivity compared to the control strain KQ02. This integrated approach addressed key bottlenecks in cadaverine biosynthesis, and offers insights applicable to other diamine production systems. These results demonstrated the exceptional efficacy of our biosensor in mining large‐scale libraries for enhanced diamine bioproduction. This work describes a genetically encoded biosensor that enables rapid pathway optimization and beneficial target discovery. We emphasize the importance of such high‐throughput tools in transitioning metabolic engineering from “local fine‐tuning” to “system‐wide reconstruction”, and anticipate this strategy will serve as a transferable blueprint for producing other value‐added compounds.

## 4 Experimental Section

### 4.1 Strains, Plasmids, and Medium

All strains and plasmids used in this study were listed in Tables S3 and S4, respectively. *E. coli Trans*1‐T1 was used to construct recombinant plasmids and BL21(DE3) was used to express protein. Plasmid extraction was performed using the TIANprep Mini Plasmid Kit (Tiangen); Isolation of genomic DNA was performed using the TIANamp Bacteria DNA Kit (Tiangen); PCR amplification was performed using the 2×Phanta Max Master Mix (Dye Plus) (Vazyme); Enzymatic digestion was performed using QuickCut (TaKaRa); Universal DNA Purification Kit (Tiangen) was used for recovery of DNA fragments from TAE agarose gel; ClonExpress II One Step Cloning Kit‐C112, C113 and C116 (Vazyme) was used for homologous recombination. Luria‐Bertani (LB) medium (10 g/L peptone, 5 g/L yeast extract, 5 g/L NaCl) was used for regular cultures of *E. coli* strains. LB medium containing different concentrations of cadaverine was used as performance characterization medium for biosensor. The lysine‐producing strain KQ01 was constructed de novo by using MG1655(DE3) as the chassis cell. The genomic integration and deletion were achieved using the CRISPR‐Cas9 system, following standard procedures for donor DNA construction, gRNA design, transformation, colony validation, and plasmid removal. Genomic multicopy integration strategy referred to Zhang et al. [56]. The fermentation medium of shake flask and 5L fermenter for cadaverine fermentation have been described in our previous work [57]. If needed, when the OD_600_ of the engineered strains in the 5 L fermenter was approximately 6, 50 µM IPTG was added for induction. If needed, the antibiotics at concentrations of 100 mg/L ampicillin, 50 mg/L chloramphenicol, 50 mg/L Kanamycin, and 80 mg/L streptomycin were added. For dual‐plasmid systems, the concentration of antibiotics was reduced to half of the regular‐used concentration, respectively. For tri‐plasmid systems, the concentration of antibiotics was reduced to one‐third of the regular‐used concentration, respectively.

### 4.2 Transcriptomic Analysis

Transcriptome analysis for strains NkdsA, NnadA, NhypB, and NrihA. Based on the production curves, the sampling time was determined to be the mid fermentation stage where the difference in cadaverine concentration was most significant. The equal amounts of cultures were centrifuge at 4°C and 4000 rpm for 10 min to collect bacterial cells. The obtained cells were washed twice with sterile water at 4°C and stored them in 1.5 mL centrifuge tubes without RNA. Finally, these two cell pellets were sent to Shanghai Paisenno Biotechnology for transcriptome sequencing analysis. The analysis work includes total RNA extraction, total RNA quality detection, rRNA removal, RNA fragmentation, cDNA synthesis, PCR enrichment library fragments, library quality inspection, and Illumina platform sequencing. The experiment was conducted in triplicate.

### 4.3 DNA Manipulation

All the primers were synthesized by Nanjing Qingke (Table S5). To construct a reporter plasmid pcadR‐GFP, the plasmids pET28a and pTrc99a were used to construct a recombinant vector plasmid, and green fluorescent protein (GFP) coding gene *gfp* was inserted into the recombinant plasmid. The specific methods to construct recombinant vector plasmid are as follows. To construct the pET28a plasmid without lacO and lacI, we used the pET28a plasmid as the template, cadR‐F as the upstream primer, and cadR‐R as the downstream primer to PCR the pET28a plasmid, obtaining the fragment pET28a (Δ *lacIO*); To construct the pcadR recombinant plasmid containing rrnB1,2 Terminator, the target fragment was obtained by PCR with pET28a (Δ lacIO) as template, 28a‐F and 28a‐R as the upstream and downstream primers, respectively. At the same time, Multiple Cloning Site (MCS) region and rrnB1,2 Terminator were obtained by PCR with pTrc99a as template, 99a‐F and 99a‐R as the upstream and downstream primers, respectively. The two fragments obtained above were digested with NcoI/XhoI and ligated by T4 Ligase to obtain the target recombinant plasmid pcadR. Finally, the plasmid pcadR was linearized by restriction endonuclease KpnI and NcoI. At the same time, the target gene *gfp* was amplified by PCR with the plasmid pTrc99a‐GFPuv preserved in the laboratory as the template, *gfp*‐F and *gfp*‐R as the upstream and downstream primers, and the recombinant plasmid pcadR‐GFP was obtained by homologous recombination (Figure S1). There are two restriction sites BglII and XbaI in the upstream of *gfp* gene. These two restriction sites are used to insert the promoters of different gene targets to facilitate the characterization of promoters.

To construct cadaverine‐responsive promoter library, the reporter plasmid pcadR‐GFP was used to characterize the promoters of differentially expressed genes. Using the *E. coli* KQ01 genome as the template, the 150 bp promoter region of each differentially expressed gene was cloned by PCR with 80 pairs of primers. The reporter plasmid pcadR‐GFP was linearized by restriction endonuclease XbaI and BglII. The recombinant plasmid pcadR‐promoter‐GFP was constructed by homologous recombination.

To obtain truncated variants of the promoter P_pyrF_, we first divided the 150‐bp sequence into upstream (−65 to −1 relative to the −35 hexamer) and downstream (+1 to +58 relative to the −10 hexamer) segments. For the truncation upstream of the −35 hexamer, we performed PCR amplification using P_pyrF_‐GFP as the template, pyrF‐10/20/30/40/50/60/70 as upstream primers and pyrF‐xia as downstream primer. These primers were designed with restriction sites for BglII and XbaI at their ends. The reporter plasmid pCadR‐GFP was linearized by double digestion with BglII and XbaI to obtain a linearized vector, which was subsequently ligated with the amplified fragments using T4 DNA ligase (Takara). The same method was applied to truncate the downstream region of the −10 hexamer yielding truncated variants of different lengths. The primer sequences were listed in Table S5.

The promoter spacer sequence between −35 and −10 hexamer was randomly mutated to construct a mutation library. The target fragment was obtained by reverse PCR amplification with plasmid P_pyrF_‐D40‐GFP as template, P_pyrF_‐jian‐F and P_pyrF_‐jian‐R as primers. The target fragment was purified and digested with restriction endonuclease DpnI. Then, the digestion products were transformed into competent cells *Trans*1‐T1 to obtain a mutation library.

To construct plasmid P_pyrF_‐F5‐GFP‐*cadA*, the vector P_pyrF_‐F5‐GFP was linearized using the restriction enzymes SalI and HindIII. The *cadA* gene was amplified by PCR using the plasmid pCDF duet‐*cadA* as a template and primers *cadA*‐GFP‐F/R. The plasmid P_pyrF_‐F5‐GFP‐*cadA* was obtained through homologous recombination.

### 4.4 Characterization of the Cadaverine Biosensor

The strains containing the cadaverine biosensor were inoculated in 5 mL LB medium with different concentrations of cadaverine and cultured at 37°C overnight for fluorescence detection. To detect the fluorescence intensity, 1 mL bacterial solution was taken, centrifuged at 12 000 rpm and 4°C for 1 min, and the supernatant was abandoned and re‐suspended with 1 mL PBS buffer solution, 200 µL of the re‐suspended solution was taken on the black 96‐well plate. The excitation wavelength of 485 nm and emission wavelength of 526 nm were set by Thermo fluorescence analyzer to measure the fluorescence intensity of GFP.

### 4.5 Protein Expression and Purification

To determine whether the protein was successfully expressed, the cells were collected by centrifugation, then washed once with deionized water and twice with PBS solution. The cell density was controlled at an OD_600_ of 30, and ultrasonic treatment was performed. The broken cells were centrifuged at 4°C for 10 min and the rotational speed was 12 000 rpm. The expression of soluble protein in the supernatant was analyzed by SDS‐PAGE, which containing 12% separated gel and 5% concentrated gel. Then the gel was stained with Coomassie brilliant blue staining solution and decolorized with decolorizing solution to observe the protein. When inducing protein of transcription factors, 0.5 mM IPTG were used.

To purify the transcription factors, the harvested cells were sonicated for 10 min on ice and centrifuged at 4°C. The supernatant was loaded onto a Ni‐NTA column and then eluted with elution buffer (20 mM Na_2_HPO_4_, 200 mM NaCl, 500 mM imidazole, pH 7.5). The collected protein solution was condensed using Amicon Ultra 15 (Millipore, MA, 5/10/30 kDa, USA) at 4000×g for 30 min.

### 4.6 Electrophoretic Mobility Shift Assay (EMSA)

The promoter region of the *E. coli pyrF* gene was analyzed using the BPROM program implemented in the SoftBerry web server (http://www.softberry.com) [24]. BPROM predicts bacterial σ^70^ promoters based on a linear discriminant function (LDF) combining promoter consensus motifs and nucleotide composition features. A 150‐bp DNA sequence upstream of the *pyrF* open reading frame (ORF) was submitted to BPROM. The prediction was performed using the default BPROM settings, including the promoter threshold value of 0.20. The predicted σ^70^ promoter elements (‐10 and ‐35 boxes), LDF score, and oligonucleotide matches corresponding to known transcription factor binding sites were obtained from the BPROM output. Only transcription factor binding sites reported by BPROM within the predicted promoter region were included for subsequent analysis. For EMSAs, the recombinant plasmids pET28a‐*argR/ihfA/ihfB/rpoD* were constructed. Taking pET28a‐*argR* as an example, the genome of *E. coli* MG1655 strain was used as template, the *argR* gene was amplified by PCR with *argR*‐F and *argR*‐R as upstream and downstream primers, and the plasmid pET28a was linearized with Vector‐F and Vector‐R as upstream and downstream primers. The recombinant plasmid pET28a‐*argR* was obtained by homologous recombination. In addition, biotin‐labeled probes were prepared by PCR using the plasmid P_pyrF_‐F5 as a template and probe‐F and probe‐R as biotin‐labeled primer pair. The DNA fragments were purified using the VAHTS DNA Clean Beads (Vazyme). DNA concentration was quantified using a NanoDrop 2000C (Thermo, USA). EMSAs were performed according to a previously described method [58]. The reaction mixture contained 50 nmol of DNA biotin‐labeled probes and various amounts of protein in the binding buffer. The mixtures were co‐incubated for 25 min at 15°C and then treated by electrophoresis on a 6% native polyacrylamide gel. The fluorescence signal was detected using a Tanon 4600 maging system.

### 4.7 IHF‐DNA Binding Analysis

To predict the DNA binding sites of IHF, the presence of sites with significant similarity to the IHF consensus sequence was evaluated by scanning the position‐specific scoring matrix (PSSM) available at the Regulon database along unmutated/mutated promoter using the program matrix‐scan from the regulatory sequence analysis tools (RSAT) web server (http://rsat.eead.csic.es/). We set weight score cut‐off of 2.0.

To investigate the alterations in binding affinity between IHF and its binding sites following mutation, we employed AlphaFold3 to predict the three‐dimensional structures of IHF‐DNA complexes. Specifically, a 24‐base pair DNA sequence encompassing the IHF binding region was utilized as input for the computational modeling of protein‐DNA interaction conformations. The top‐ranked model was selected based on the highest predicted pLDDT (per‐residue confidence score) and ipTM (interface predicted TM‐score). The protein‐DNA complex structure predicted by AlphaFold3 was further optimized using FoldX (version 5.0). Briefly, the predicted structural model was subjected to the RepairPDB command of FoldX to refine side‐chain conformations, hydrogen bond networks, and eliminate steric clashes. Subsequently, the refined structure was used to calculate the protein‐DNA interaction energy via the AnalyseComplex command in FoldX. The interaction energy between protein and DNA was computed by defining appropriate chain identifiers corresponding to each component of the complex.

MD simulations were conducted starting from the protein‐DNA complex structure predicted by AlphaFold3. The complex was solvated within a TIP3P water box, defined as a cubic box extending 10 Å from the IHF protein center. The system was neutralized and adjusted to an ionic strength of 0.2 M using K^+^ and Cl^−^ ions. Protein and DNA interactions were described using the ff14SB and BSC1 force fields, respectively. MD simulations were performed for 150 ns under constant temperature (300 K) and pressure (1 atm) conditions, following standard simulation protocols.

### 4.8 Optimization of Transporter cadB Expression Level

To regulate *cadB* expression in cadaverine‐producing strains, the recombinant plasmid P_pyrF_‐H12/D2/E1/F5/A3/C8/G4‐GFP was used as template, and ΔGFP‐F and ΔGFP‐R were used as primers for PCR amplification to linearize the vector. At the same time, the target fragment of *cadB* was obtained by PCR using *E. coli* KQ01 genome as template and *cadB*‐F and *cadB*‐R as primers. Finally, the recombinant plasmid P_pyrF_‐H12/D2/E1/F5/A3/C8/G4‐*cadB* was constructed by homologous recombination. Then, the resistance gene and the replicon of the cadaverine biosensor P_pyrF_‐F5‐GFP were replaced by Cm^R^ and the RSF ori, respectively. Finally, the recombinant plasmid P_pyrF_‐H12/D2/E1/F5/A3/C8/G4‐*cadB* and cadaverine biosensor CR‐P_pyrF_‐F5‐GFP were introduced into the cadaverine producing strain KQ02 to construct the *cadB* expression library. The obtained strains were cultivated in fermentation medium for 48 h at 37°C, and the fluorescence intensity and corresponding cadaverine concentration were measured.

### 4.9 Construction of Bacterial Artificial Chromosome (BAC) Plasmid with LoxPsym Locus

BAC refers to a bacterial chromosome cloning vector constructed based on F‐plasmid. It is commonly used to clone DNA fragments of about 150 kb in size and can store up to 300 kb base pairs. LoxPsym sites were inserted into both ends of each gene via circular polymerase chain reaction (PCR). Homologous terminal fragments (approximately 25 base pairs) were appended to the termini of these loxPsym‐flanked fragments using PCR. Employing homologous recombination, the resulting constructs were seamlessly integrated onto the pBeloBAC11 plasmid vector utilizing the seamless cloning kit (Bomard). This process was iterative, with two to three DNA fragments being assembled at a time, until all genes were successfully assembled. Each gene contains specific 100 bp sequences to detect the structural variation.

### 4.10 Construction of in Vitro SCRaMbLEd Library and Strain Screening Using the Biosensor‐Based HTS Platform

The Cre recombinase reaction was set up as per the manufacturer's instructions (NEB, M0298L) and incubated at 37°C for 1 h. The Cre enzyme was heat inactivated for 10 min at 70°C. For top‐down in vitro SCRaMbLE, 100 ng of DNA was added in a total reaction volume of 10 µL with 1 µL of Cre recombinase. The SCRaMbLEd pools were transformed to hosts for genotype and phenotype testing. The rearranged plasmid was recovered from E. coli NEB10‐beta. The 5 mL of overnight cultured cells was collected and the BAC/PAC large plasmid extraction kit was used to recover the SCRaMbLEd libraries. The recovered plasmids were analyzed by restriction endonuclease analysis and gene specific primer PCR.

The two SCRaMbLEd pathway libraries were transferred into lysine producing strain KQ01 with cadaverine biosensor and lysine decarboxylase, and SCRaMbLEd strain libraries were obtained. The difference between the control strain and SCRaMbLEd strain libraries was that the former contained the plasmid without rearrangement. Given the positive role of IHF in enhancing cellular tolerance, and to eliminate potential interference from gene interactions, we chose the P_pyrF_‑F5‑GFP sensor lacking the Ihfβ optimization module for both the SCRaMbLE and ASKA screenings (Figure S28). After plate culture, the mixed bacteria were transferred to seed medium and cultured at 37°C for 10 h. Then, the seed solution was transferred to the fermentation medium without calcium carbonate at 5% inoculum, and 20 g/L glucose and 10 g/L ammonium sulfate were supplemented when the glucose was about to run out. At the end of fermentation (48 h), sterile PBS was used to control the final OD_600_ of the culture to 0.2∼0.4, and the solution is collected into sterile flow cytometry tubes for FACS. Screening was performed using the MoFlo XDP flow cytometer sorter (Beckman Coulter, Inc., Fullerton, CA, USA) according to the method described earlier [59]. To improve the positive rate, flow cytometry purification model was used for sorting. The isolated cells were cultured in a 96‐well culture plate containing 200 µL seed medium, then the seed solution was inoculated into 800 µL fermentation medium. The fluorescence intensity was measured by Thermo fluorescence analyzer.

### 4.11 High‐Throughput Screening of a Genome‐Scale Overexpression Library

The ASKA library contains 4,123 strains, each carrying a single‐gene overexpression plasmid with pCA24N as the vector [60]. To obtain the ASKA plasmid library, strains from a 96‐well plate were grouped. Using a multichannel pipette, 10 µL of bacterial culture from each well was transferred to 10 mL of LB liquid medium containing 25 µg/mL chloramphenicol, then incubated with shaking for 12 h. Plasmid extraction was performed per plate, with each plate corresponding to a tube of ASKA library plasmids. 2 µL plasmid of each tube were co‐transformed with plasmid P_pyrF_‐F5‐GFP‐*cadA* into KQ01 cells. The control strain contains the empty vector pCA24N and plasmid P_pyrF_‐F5‐GFP‐*cadA*. After single colonies appeared on plates, they were picked for flask fermentation (with 12.5 µg/mL chloramphenicol and 12.5 µg/mL kanamycin). When OD_600_ reached around 0.6, 50 µM IPTG was added. When glucose was depleted, glucose and ammonium sulfate were supplemented in two batches. After fermentation, samples were collected using the same method as before, totaling 57 tubes. The cell sorting method was the same as previously described. The screened strains were validated via flask fermentation, and strains with high cadaverine production were sequenced. The target genes were determined through BLAST analysis on the NCBI website.

### 4.12 Construction and Characterization of a Multi‐Gene Combinatorial Library

The library was constructed through a three‐fragment random homologous recombination method (Figure 6A). The target fragments to be amplified were designated as Region I, Region II, and Region III. The primers for Region I were P‐I‐F/P‐I‐R, for Region II were P‐II‐F/P‐II‐R, and for Region III were P‐III‐F/P‐III‐R. PCR amplification was performed using four plasmids, pCA24N‐*kdsA* (957 bp), pCA24N‐n*adA* (1146 bp), pCA24N‐*hypB* (976 bp), and pCA24N‐*rihA* (1038 bp), as templates. The expression vector pTrc99a was linearized by inverse PCR using primers M‐F/M‐R. A multi‐gene combinatorial library was obtained through random homologous recombination. For the construction of the control plasmids, the vector pTrc99a was linearized in the same manner. Using the primers P‐I‐F/P‐III‐R, four fragments were amplified by PCR from the four plasmids (pCA24N‐*kdsA*, pCA24N‐n*adA*, pCA24N‐*hypB*, and pCA24N‐*rihA*). These fragments were then subjected to homologous recombination reactions with the linearized vector, respectively. The control plasmids pTrc99a‐*kdsA*, pTrc99a‐*nadA*, pTrc99a‐*hypB*, and pTrc99a‐*rihA* were then obtained.

To screen and characterize the multi‐gene combination library, the mixed plasmids from the library and the biosensor P_pyrF_‐F5‐GFP‐*cadA* were transformed into KQ01. The control plasmids pTrc99a, pTrc99a‐*kdsA*, pTrc99a‐*nadA*, pTrc99a‐*hypB*, and pTrc99a‐*rihA* were transformed with biosensor P_pyrF_‐F5‐GFP‐*cadA* into KQ01 to obtain the control strains. Three hundred single colonies (approximately five times coverage) were randomly picked from the plates and inoculated into 96‐well shallow plates for seed culture for 12–16 h. The seed cultures were then transferred at a 2% inoculum volume into the fermentation medium in 96‐well deep plates, using the same cultivation method as described above. Positive samples with higher GFP fluorescence intensity and GFP/OD_600_ values were selected for subsequent shake‐flask re‐screening experiments. Several samples with higher cadaverine concentrations were identified, and their multi‐gene combinatorial patterns were determined by sequencing.

### 4.13 Crystal Violet Staining Assay

The biofilm formation ability of strains NkdsA, NnadA, NhypB, NrihA, and KQ02 was evaluated. Each strain was cultured in shake‑flask seed medium to an OD_600_ of 1.0, and 20 µL of the culture was inoculated into 96‑well plates containing shake‑flask fermentation medium. Six replicate wells were set for each strain, along with blank control wells containing medium only. The plates were incubated at 37°C, and samples were taken at 36 and 48 h for biofilm quantification. After incubation, the culture medium was gently aspirated to avoid disrupting the biofilm. The wells were washed three times with sterile PBS (3–5 min per wash) to remove non‑adherent cells and residual medium, followed by fixation with 4% paraformaldehyde for 15 min. After removing the fixative, 200 µL of 0.1% crystal violet solution was added to each well and stained for 15–20 min at room temperature. The wells were then rinsed with sterile water 3–5 times until the washings became clear. To solubilize the retained crystal violet, 200 µL of 33% acetic acid was added to each well, and the plate was shaken for 10–15 min. Finally, the absorbance at 570 nm was measured using a microplate reader. The relative absorbance was calculated by subtracting the blank control values, and the resulting OD_570_ values were used to indirectly reflect biofilm biomass.

### 4.14 Cell Mortality Rate Assay

Membrane integrity was quantified by SYTO 9 and propidium iodide (PI) staining followed by flow‐cytometric analysis. 24 h fermentation broth was harvested by centrifugation (4 500 rpm, 3 min, 4°C) and the pellet was washed twice with ice‐cold PBS. Approximately 1 × 10^6^ cells were re‐collected by centrifugation, resuspended in Assay buffer containing PI at a final concentration of 10 µg/mL and SYTO 9 at a final concentration of 10 µM, and adjusted to a cell density of 1×10^6^ cells/mL. After 20 min incubation at 37°C in the dark, cells were pelleted (4 500 rpm, 3 min, 4°C), washed twice with ice‐cold PBS, and finally resuspended in PBS at OD_600_ of 0.01 to 0.1. Flow‐cytometric acquisition was performed on a Beckman CytoFLEX equipped with a 488 nm excitation laser and a 525 nm (green)/575 nm (red) emission filter; 30 000 cells per sample were recorded and analysed using FlowJo software. Gating boundaries for intact cells were established with the plasmid‐free reference strain KQ01 and subsequently applied to all experimental samples to calculate the percentage of dead cells.

### 4.15 Culture Conditions and Analytical Methods

For cadaverine fermentation, a fresh single colony of the strain or mixed bacteria were inoculated into the 5 mL of shaking tube seed medium and cultured for 10 h at 37°C. An aliquot of 1 mL of the overnight culture was inoculated into 30 mL shake flask fermentation medium, and the culture was induced at 37°C for 48 h. The biomass was monitored by measuring OD_600_. The concentration of lysine and glucose was measure by an SBA‐40c biosensor analyzer (Shandong Province Academy of Sciences, China) [23]. The cadaverine was analyzed by High Performance Liquid Chromatography (HPLC) [23, 61]. HPLC analysis of cadaverine was performed on an Agilent 1290 Infinity system (Agilent Technologies, Santa Clara, CA, USA) equipped with a fluorescence detector (FLD G1321B). Specific steps were conducted according to our previous study [62]. The intracellular ATP levels were measured according to the instructions provided with the ATP Assay Kit from Beyotime. To maintain the stability of ATP, ultrasonic cell lysis was performed on ice. Similarly, the intracellular levels of NAD^+^ and NADH were measured according to the instructions provided with the NAD^+^/NADH Assay Kit from Beyotime.

### 4.16 For Determination of Intracellular Cadaverine Concentrations

Cells (10 OD_600_) were harvested, washed three times with 5 mL ice‐cold PBS (Na_2_HPO_4_ 11.1 g/L, NaH_2_PO_4_ 2.97 g/L, pH 7.0) at 4000 rpm for 5 min at 4°C, and snap‐frozen in liquid nitrogen. The pellet was resuspended in 1 mL PBS, sonicated, and centrifuged at 12 000 rpm for 10 min at 4°C. Proteins were precipitated by adding 1/10 (v/v) of ice‐cold 100% trichloroacetic acid, followed by vortexing, 15 min incubation on ice, and centrifugation. The supernatant was analyzed by HPLC to determine the intracellular cadaverine concentration.

### 4.17 Statistical Analysis

All data were processed and analyzed in GraphPad Prism. The error bar represents the standard deviation of the mean. The number of copies was given in the legend, and all copies were included in the manuscript. All graphs, bars, and dose‐response functions displayed were generated in GraphPad Prism. In the statistical analysis, multiple t tests‐one per row were performed and *p* < 0.05 was considered statistically significant.

## Author Contributions

**Jia Feng**: conceptualization, formal analysis, investigation, methodology, validation, writing – original draft. **Getao Liu, Ding Ma, Jiaxiang Yuan, Xinyi Zhang**: conceptualization, methodology, validation. **Weihao Wang, Chen Ma**: formal analysis, investigation, methodology. **Yang Liao, Siyuan Gao**: investigation, validation. **Lei Qin**: formal analysis, resources. **Jiao Feng, Sheng Xu**: supervision, project administration, funding acquisition. **Xin Wang, Kequan Chen**: conceptualization, methodology, writing – review & editing, supervision, project administration, funding acquisition.

## Conflicts of Interest

The authors declare no conflicts of interest.

## Supporting information




**Supporting File**: advs77720‐sup‐0001‐SuppMat.docx.

## Data Availability

The data that support the findings of this study are available from the corresponding author upon reasonable request.
