# Supplementary material for: Design of a Novel Diamine Biosensor to Guide the Engineering of Escherichia coli for Cadaverine Overproduction
Source: Adv Sci (Weinh). 2026 Sep 27:e77720. Online ahead of print. doi: 10.1002/advs.77720 (PMC13616330; doi:10.1002/advs.77720)
Supplement: Supplementary file 1 [file ADVS-9999-e77720-s001.docx]

Supporting Information

**Design of a Novel Diamine Biosensor to Guide the Engineering of *Escherichia coli* for Cadaverine Overproduction**

*Jia Feng, Getao Liu, Ding Ma, Jiaxiang Yuan, Xinyi Zhang, Weihao Wang, Chen Ma, Yang Liao, Siyuan Gao, Lei Qin, Jiao Feng, Sheng Xu, Xin Wang*, and Kequan Chen**

**Supplementary figures**

**Figure S1.** Schematic diagram of the reporter plasmid construction. The 100 cadaverine-responsive promoters were cloned to control GFP expression.

**Figure S2.** Fluorescence intensity of promoter P_pyrF_ and P_grcA_ response to different cadaverine concentrations.

**Figure S3.** Response of promoter P_pyrF_ in *ihfB or ihfA* overexpression strain and *ihfB-ihfA* co-overexpression strain. *ihfB* overexpression reduced basal P_pyrF_ activity by 51.1% while preserving cadaverine-dependent induction. In contrast, *ihfA* overexpression reduced the induction fold, and co-overexpression of *ihfA* and *ihfB* further reduced the fluorescence response. Data repressent mean ± SD of three biological replicates.

**Figure S4.** SDS-PAGE assay of four transcription factors. Lane 1, 3, 5, and 7 represent the soluble proteins, and lane 2, 4, 6 and 8 represent the inclusion bodies.

**Figure S5.** The binding of the Ihfβ or the mixed Ihfα/Ihfβ samples to promoter DNA with or without cadaverine addition was characterized based on EMSA. In the absence of cadaverine, the mixed Ihfα/Ihfβ samples had specific DNA binding activity on the probe DNA (Lane 2). In the presence of 5 g/L cadaverine,the mixed Ihfα/Ihfβ samples and the Ihfβ had no specific DNA binding activity on the probe DNA (Lanes 3 and 5). In the presence of 20 g/L cadaverine, the mixed Ihfα/Ihfβ samples had no specific DNA binding activity on the probe DNA (Lane 4).

**Figure S6.** Comparison of RMSF Between the heterodimer Ihfαβ-24 (24-bp Unmutated Promoter) complex and the homodimer Ihfβ-24 (24-bp Unmutated Promoter) complex (Left); Comparison of RMSF Between the heterodimer Ihfαβ-50 (50-bp Unmutated Promoter) complex and the homodimer Ihfβ-50 (50-bp Unmutated Promoter) complex (Right).

**Figure S7.** RMSF analysis of the Ihfα-cadaverine complex, Ihfβ-cadaverine complex and Ihfαβ-cadaverine complex.

**Figure S8.** The binding of the Ihfβ or the mixed Ihfα/Ihfβ to promoter DNA with diamine (1,4-butanediamine or 1,6-hexanediamine) addition was characterized based on EMSA. At 20 g/L 1,4-butanediamine (Lanes 3 and 5) or 1,6-hexanediamine (Lanes 4 and 6), the Ihfβ homodimer (Lanes 3 and 4) or the mixed Ihfα/Ihfβ (Lanes 5 and 6) had no specific DNA binding activity on the probe DNA, and the controls were shown in lanes 1, 2.

**Figure S9.** Response of the promoter P_pyrF_ to different concentrations of compounds. (A) Response of the promoter P_pyrF_ to three amino acids (lysine, arginine, histidine). (B) Response of the promoter P_pyrF_ to two polyamines (spermine, spermidine). (C) Response of the promoter P_pyrF_ at pH 7 to 9 in the absence of exogenous amines. Data represent mean ± SD of three biological replicates.

**Figure S10.** Screening of mutant promoter library under stress of 20 g/L cadaverine. A mutation library was generated by randomizing the 15-bp spacer sequence in P_pyrF_-D40. The mutant library was then transformed into *E. coli* and screened via fluorescence assays in medium supplemented with 20 g/L. Data represent mean ± SD of three biological replicates.

**Figure S11.** GC content in mutant spacer sequences. Sequencing of seven mutants revealed that its spacer GC content increased from 40% to 80%.

**Figure S12.** Structure of IHFα/β heterodimer. (A) Experimental structure from PDB 1IHF; (B) Structure predicted by AlphaFold3; (C) Comparison between experimental structure and predicted structure. The experimental results were aligned with the predicted structure, and the Root Mean Square Deviation (RMSD) between the two structures was calculated to be 0.33 Å, indicating that the two structures are nearly identical at the atomic level, with differences arising only from minor conformational fluctuations in flexible regions.

**Figure S13.** Structure of mutant/unmutant promoter sequence. (A) Unmutant promoter. (B) Mutant promoter. The structure of mutant/unmutant promoter sequence was prepared by nucleic-acid modeling tool 3DNA.

**Figure S14.** Measurement of intracellular and extracellular cadaverine production during shake‑flask fermentation of the strain with dynamic regulation of CadB.

**Figure S15.** Time courses of *cadB* transcription, intracellular cadaverine accumulation and biosensor response in the dynamically regulated strain P_pyrF_-G4-cadB. (A) *cadB* transcription levels in the strain P_pyrF_-G4-cadB during shake-flask fermentation, determined by RT-qPCR at 12, 24, and 36 h. Transcript levels were normalized to 16S rRNA and expressed as log_2_(fold change) relative to the 0 h sample of each strain. (B) Intracellular cadaverine titers of the corresponding cultures at the same time points. (C) Biosensor response of the corresponding cultures at the same time points. Data are mean ± SD of three independent biological replicates.

**Figure S16.** Comparison of promoter output strength between the constitutive Trc promoter and the cadaverine-responsive promoter P_pyrF_-G4.

**Figure S17.** Feasibility analysis of *in vitro* SCRaMbLE using Cre recombinase. (A) Gel electrophoresis analysis of an *in vitro* SCRaMbLEd library. Gel electrophoresis of the linearized library revealed diverse DNA bands, indicating high efficiency and diversity of the SCRaMbLEd pathway library. (B) Unique pathway structures of Module 1 SCRaMbLEd pathway library. (C) Unique pathway structures of Module 2 SCRaMbLEd pathway library. Sequencing of randomly selected clones confirmed the occurrence of rearrangement events in both modules.

**Figure S18.** Fluorescence intensity and cadaverine production of Module 1 and Module 2 SCRaMbLEd strain libraries in mixed-feed shake-flask fermentation. The two SCRaMbLEd pathway libraries were transformed into a lysine producing strain KQ01 harboring cadaverine biosensor and lysine decarboxylase, yielding SCRaMbLEd strain libraries of M1 and M2. Both the M1 and M2 libraries exhibited higher fluorescence intensity and cadaverine production than the control strain MC. Data represent mean ± SD of three biological replicates.

**Figure S19.** Fluorescence-activated cell sorting of rearrangement strains. (A) Flow cytometry analysis of Module 1 SCRaMbLEd strains library. (B) Flow cytometry analysis of Module 2 SCRaMbLEd strains library. Fluorescence-activated cell sorting (FACS) was performed on the fermentation broth of two SCRaMbLEd library, successfully isolating single cells with high fluorescence intensity.

**Figure S20.** Screening of SCRaMbLEd strains for high cadaverine production using cadaverine biosensor. (A) GFP/OD_600_ values for rescreening of SCRaMbLEd strains. This process yielded five improved strains from Module 1 and eight from Module 2 SCRaMbLEd strains, all showing significantly higher fluorescence than the control. (B) Correlation of cadaverine concentration with biosensor fluorescence outputs, a straight-line curve was fitted using GraphPad Prism linear regression fit, R^2^ = 0.91.

**Figure S21.** Fluorescence-activated cell sorting of an ASKA library combining with the biosensor. Following induction and cultivation, cells with elevated fluorescence were isolated, and overexpressed genes in selected clones were subsequently identified by sequencing and BLAST analysis, allowing for uncovering candidate genes that potentially contributed to enhanced cadaverine production.

**Figure S22.** Cadaverine production of the strains KQ02, NkdsA, NnadA, NhypB, and NrihA in fed-batch fermentation. To elucidate the metabolic influence of these four genes during the cadaverine production, we constructed strains overexpressing each gene targets (*kdsA*, *hypB*, *rihA*, and *nadA*) for fed-batch fermentation, and performed transcriptomic analysis on samples taken at mid fermentation stage. Data represent mean ± SD of three biological replicates.

**Figure S23.** Volcano plot of differential expression. Comparative analysis revealed that all four engineered strains exhibited significantly transcriptional changes relative to the control strain KQ02.

**Figure S24.** KEGG enrichment analysis. KEGG pathway enrichment analysis highlighted biofilm formation as a common upregulated pathway across all four engineered strains. Conversely, analysis of downregulated pathways identified the tricarboxylic acid (TCA) cycle as a commonly suppressed pathway across all strains.

**Figure S25.** Biofilm formation of strains NkdsA, NnadA, NhypB, NrihA, and control KQ02 was assessed by crystal violet staining. Three biological replicates; Error bars represent standard deviations. *P value< 0.05, **P value < 0.01, ***P value < 0.001, and NS indicates not significant as determined by t-test.

**Figure S26.** Intracellular NADH and NAD^+^ content in each engineered strain. Measurements of intracellular NAD^+^ and NADH revealed that the four genes altered the level of NAD⁺ and NADH. Data represent mean ± SD of three biological replicates.

**Figure S27.** Recombination and integration verification. From right to left: Marker; Band 1: 3456 bp; Band 2: 3456 bp; Band 3: 3456 bp; Band 4: 2971 bp; Band 5: 2394 bp; Band 6: 2520 bp; Band 7: 3004 bp; Band 8: 1293 bp; Band 9: 3456 bp; Band 10: 30 04 bp; Band 11: 3267 bp. Analysis of the positive clones revealed that three-fragment assemblies accounted for 81.8%, with two-fragment and single-fragment assemblies accounting for 9.1% each.

**Figure S28.** Overexpression of IHF improved strain growth under cadaverine stress.

**Supplementary tables**

**Table S1.** Predicting the DNA binding sites of IHF.

| seq_id | ft_  type | ft_  name | strand | start | end | sequence | weight | Pval | ln_  Pval | sig | rank | rank_  pm |
| --- | --- | --- | --- | --- | --- | --- | --- | --- | --- | --- | --- | --- |
| unmutant | site | WWWTMADTHHDTTA | R | -85 | -72 | AAATAAAAAATTAA | 5.1 | 8.60e-04 | -7.057 | 3.065 | 1 | 1 |
| unmutant | site | WWWTMADTHHDTTA | D | -87 | -74 | TGTTAATTTTTTAT | 3.6 | 3.70e-03 | -5.601 | 2.433 | 2 | 2 |
| unmutant | site | WWWTMADTHHDTTA | D | -88 | -75 | CTGTTAATTTTTTA | 3.5 | 4.10e-03 | -5.504 | 2.39 | 3 | 3 |
| unmutant | site | WWWTMADTHHDTTA | R | -87 | -74 | ATAAAAAATTAACA | 3.1 | 5.70e-03 | -5.167 | 2.244 | 5 | 5 |
| unmutant | site | WWWTMADTHHDTTA | D | -95 | -82 | ATCGCAACTGTTAA | 3.1 | 5.70e-03 | -5.167 | 2.244 | 4 | 4 |
| mutant | site | WWWTMADTHHDTTA | D | -95 | -82 | ATCGCAACTGTTAA | 3.1 | 5.70e-03 | -5.167 | 2.244 | 1 | 1 |
| unmutant | site | WWWTMADTHHDTTA | R | -84 | -71 | CAAATAAAAAATTA | 2.7 | 7.90e-03 | -4.839 | 2.101 | 6 | 6 |

**Table S2.** RBS sequences for optimizing the expression strength of the mutant transcription factor.

| Promoter | RBS sequence | Translation Initiation Rate (a.u.) |
| --- | --- | --- |
| Trc | TAAGTAGAGGTTCCT | 18826 |
| Trc | GGAATTAAGGAGATA | 11839 |
| Trc | CTACCTACGAGGTTATCTC | 7581 |
| Trc | AGATATAGGGACGTTAT | 4596 |

**Table S3.** List of *E. coli* strains used in this study.

| Strains | Characteristics | Reference |
| --- | --- | --- |
| *E. coli* *Trans* 1-T1 | F^–^ φ80 (*lacZ*) ΔM15 Δ*lacX*74 *hsdR* (*r_k_^–^m_k_^+^*) Δ*recA*1398 *endA*1 *tonA* | Transgen |
| BL21(DE3) | F_ ompT hsdSB (rB_mB_) dcm gal (DE3) | Transgen |
| MG1655(DE3) | K-12 F^–^ λ^–^ *ilvG*^–^ *rfb*-50 *rph*-1 (DE3) | Lab stock |
| lysine-producing *E. coli* KQ01 | *E. coli* MG1655(DE3) Δ*pfkA*, Δ*metB*, *thrA* (G477D), IS1 loci :: P_trc_-*ppc* :: P_trc_-*aspC* :: P_trc_-*lysC* (G323D/M318I) :: P_trc_-*asd* :: P_trc_-*dapA* (H118Y) :: P_trc_-*dapB* :: P_trc_-*ddh* :: P_trc_-*pntA* :: P_trc_-*pntB* :: Plac-*prk* :: P_trc_-*cbbm* :: P_ara_-*GroESL* | Lab stock  ^[18, 62-66]^ |
| *E. coli* KQ02 | *E. coli* KQ01 derivate, containing pCDF duet-*cadA* | This study |
| *cadB* library | *E. coli* KQ02 containing P_pyrF_/P_pyrF_-H12/D2/E1/F5/A3/C8/G4-*cadB*, and CR-P_pyrF_-F5-GFP | This study |
| *E. coli* CB01 | *E. coli* KQ02 derivate, containing trc-*cadB* | This study |
| *E. coli* B01 | *E. coli* KQ02 derivate, containing P_pyrF_-G4-*cadB* | This study |
| MC | *E. coli* KQ01 containing pCDF duet-*cadA*, pBeloBAC11, and P_pyrF_-F5-GFP | This study |
| M1 | *E. coli* KQ01 containing pCDF duet-*cadA*, BAC-M1, and P_pyrF_-F5-GFP | This study |
| M2 | *E. coli* KQ01 containing pCDF duet-*cadA*, BAC-M2, and P_pyrF_-F5-GFP | This study |
| MC0 | *E. coli* KQ01 containing pCDF duet-*cadA*, pBeloBAC11 | This study |
| M11 | M1 structural variant, obtained by FACS, without P_pyrF_-F5-GFP | This study |
| M12 | M1 structural variant, obtained by FACS, without P_pyrF_-F5-GFP | This study |
| M13 | M1 structural variant, obtained by FACS, without P_pyrF_-F5-GFP | This study |
| M14 | M1 structural variant, obtained by FACS, without P_pyrF_-F5-GFP | This study |
| M15 | M1 structural variant, obtained by FACS, without P_pyrF_-F5-GFP | This study |
| M21 | M2 structural variant, obtained by FACS, without P_pyrF_-F5-GFP | This study |
| M22 | M2 structural variant, obtained by FACS, without P_pyrF_-F5-GFP | This study |
| M23 | M2 structural variant, obtained by FACS, without P_pyrF_-F5-GFP | This study |
| M24 | M2 structural variant, obtained by FACS, without P_pyrF_-F5-GFP | This study |
| M25 | M2 structural variant, obtained by FACS, without P_pyrF_-F5-GFP | This study |
| M26 | M2 structural variant, obtained by FACS, without P_pyrF_-F5-GFP | This study |
| M27 | M2 structural variant, obtained by FACS, without P_pyrF_-F5-GFP | This study |
| M28 | M2 structural variant, obtained by FACS, without P_pyrF_-F5-GFP | This study |
| M2101 | *E. coli* M21 derivate, containing P_pyrF_-G4-*cadB* | This study |
| M1521 | *E. coli* KQ02 derivate, containing BAC-M3 | This study |
| M152101 | *E. coli* M1521 derivate, containing P_pyrF_-G4-*cadB* | This study |
| NkdsA | *E. coli* KQ01 derivate, containing pCDF duet-*cadA-kdsA* | This study |
| NnadA | *E. coli* KQ01 derivate, containing pCDF duet-*cadA-nadA* | This study |
| NhypB | *E. coli* KQ01 derivate, containing pCDF duet-*cadA-hypB* | This study |
| NrihA | *E. coli* KQ01 derivate, containing pCDF duet-*cadA-rihA* | This study |
| NKNR | *E. coli* KQ01 derivate, containing pCDF duet-*cadA-kdsA-nadA-hypB* | This study |
| M301 | *E. coli* KQ01 Δ*yciQ* :: P_pyrF_-G4-*cadB* :: P_trc_-*kdsA-*P_trc_*-nadA-hypB* :: P_trc_-*gapN* :: P_trc_-*cadA* :: P_trc_-*aspC* :: P_trc_-*asd* :: P_trc_-*dapA* | This study |

Abbreviations: pfkA, 6-phosphofructokinase I; ppc, phosphoenolpyruvate carboxylase; aspC, aspartate aminotransferase; lysC, aspartate kinase III; asd, aspartate-semialdehyde dehydrogenase; dapA, 4-hydroxy-tetrahydrodipicolinate synthase; dapB, 4-hydroxy-tetrahydrodipicolinate reductase; ddh, diaminopimelate dehydrogenase from Corynebacterium glutamate; pntA, NAD(P) transhydrogenase subunit alpha; pntB, NAD(P) transhydrogenase subunit beta; metB, Cystathionine gamma-synthase; thrA, fused aspartate kinase/homoserine dehydrogenase 1; prk, phosphoribulate kinase from Spinacea oleracea; cbbm, ribulose-1,5-bisphosphate carboxylase/oxygenase (RuBisCO) from Rhodospirillum rubrum; GroSL, 10 kDa chaperonin GroS and 60 kDa chaperonin GroL with Arabinose-inducible promoter; cadA, lysine decarboxylase; cadB, cadaverine transporter; kdsA, 3-deoxy-D-manno-octulosonic acid 8-phosphate synthase; nadA, quinolinate synthase; hypB, hydrogenase isoenzymes nickel incorporation protein; rihA, pyrimidine-specific ribonucleoside hydrolase; BAC, bacterial artificial chromosome.

**Table S4.** List of plasmids used in this study.

| Plasmids | Characteristics | Reference |
| --- | --- | --- |
| pTrc99a | pBR322 ori, *Amp^R^*, trc promoter, trcRBS, lacI, vector for gene expression in *E. coli* | Novagen |
| pET28a | pBR322 ori, *Kan^R^*, T7 promoter, T7RBS, lacI, vector for protein expression | Novagen |
| pBeloBAC11 | Cm*^R^*, vector for assemble genes related to cadaverine production | Miaoling |
| pCA24N | pMB1 ori, lacI gene, Cm^R^, T5 promoter | ^[59]^ |
| pTrc99a-GFPuv | *Amp^R^*, pTrc99a containing *gfp* gene | Lab storage |
| pcadR-GFP | pTrc99a and pET28a derivate, containing *gfp* gene | This study |
| P_pyrF_-GFP | pcadR-GFP derivate, containing the promoter of *pyrF* | This study |
| P_pyrF_-D40-GFP | P_pyrF_-GFP derivate with 40-bp truncation in the downstream region of promoter P_pyrF_ | This study |
| P_pyrF_-H12-GFP | P_pyrF_-D40-GFP derivate, obtained by random mutation of promoter spacer sequence | This study |
| P_pyrF_-A3-GFP | P_pyrF_-D40-GFP derivate, obtained by random mutation of promoter spacer sequence | This study |
| P_pyrF_-D2-GFP | P_pyrF_-D40-GFP derivate, obtained by random mutation of promoter spacer sequence | This study |
| P_pyrF_-C8-GFP | P_pyrF_-D40-GFP derivate, obtained by random mutation of promoter spacer sequence | This study |
| P_pyrF_-E1-GFP | P_pyrF_-D40-GFP derivate, obtained by random mutation of promoter spacer sequence | This study |
| P_pyrF_-G4-GFP | P_pyrF_-D40-GFP derivate, obtained by random mutation of promoter spacer sequence | This study |
| P_pyrF_-F5-GFP | P_pyrF_-D40-GFP derivate, obtained by random mutation of promoter spacer sequence | This study |
| pET28a-*ihfA* | pET28a derivate, expressing *ihfA* gene | This study |
| pET28a-*ihfB* | pET28a derivate, expressing *ihfB* gene | This study |
| pCDF duet-trc-*ihfB* | pCDF duet derivate, expressing *ihfB* gene | This study |
| pET28a-*argR* | pET28a derivate, expressing *argR* gene | This study |
| pET28a-*rpoD* | pET28a derivate, expressing *rpoD* gene | This study |
| pCDF duet-*cadA* | pCDF duet derivate, expressing *cadA* gene | This study |
| P_pyrF_-H12-*cadB* | P_pyrF_-H12-GFP derivate, expressing *cadB* gene | This study |
| P_pyrF_-A3-*cadB* | P_pyrF_-A3-GFP derivate, expressing *cadB* gene | This study |
| P_pyrF_-D2-*cadB* | P_pyrF_-D2-GFP derivate, expressing *cadB* gene | This study |
| P_pyrF_-C8-*cadB* | P_pyrF_-C8-GFP derivate, expressing *cadB* gene | This study |
| P_pyrF_-E1-*cadB* | P_pyrF_-E1-GFP derivate, expressing *cadB* gene | This study |
| P_pyrF_-G4-*cadB* | P_pyrF_-G4-GFP derivate, expressing *cadB* gene | This study |
| P_pyrF_-F5-*cadB* | P_pyrF_-F5-GFP derivate, expressing *cadB* gene. | This study |
| trc-*cadB* | P_pyrF_-G4-*cadB* derivate, the promoter P_pyrF_-G4 was replaced by promoter trc | This study |
| CR-P_pyrF_-F5-GFP | P_pyrF_-F5-GFP derivate. The resistance gene and the replicon were replaced by Cm*^R^* and the RSF ori, respectively. | This study |
| BAC-M1 | pBeloBAC11 derivate, expressing genes of *zwf*, *pgl*, *tkt*, *tal*, *gapN* | This study |
| BAC-M2 | pBeloBAC11 derivate, expressing genes of *ppc*, *cadA*, *lysA*, *aspC*, *lysC*, *asd*, *dapA*, *dapB*, *ddh* | This study |
| BAC-M3 | pBeloBAC11 derivate, expressing genes of *cadA*, *aspC*, *asd*, *dapA*, *gapN* | This study |
| P_pyrF_-F5-GFP-*cadA* | P_pyrF_-F5-GFP derivate, containing *cadA* gene | This study |
| pCA24N-*kdsA* | pCA24N derivate, expressing gene *kdsA* | This study |
| pCA24N-*nadA* | pCA24N derivate, expressing gene *nadA* | This study |
| pCA24N-*hypB* | pCA24N derivate, expressing gene *hypB* | This study |
| pCA24N-*rihA* | pCA24N derivate, expressing gene *rihA* | This study |
| pCDF duet-*cadA-kdsA* | pCDF duet-*cadA* derivate, expressing gene *kdsA* | This study |
| pCDF duet-*cadA-nadA* | pCDF duet-*cadA* derivate, expressing gene *nadA* | This study |
| pCDF duet-*cadA-hypB* | pCDF duet-*cadA* derivate, expressing gene *hypB* | This study |
| pCDF duet-*cadA-rihA* | pCDF duet-*cadA* derivate, expressing gene *rihA* | This study |
| pTrc99a-*kdsA* | pTrc99a derivate, expressing gene *kdsA* | This study |
| pTrc99a-*nadA* | pTrc99a derivate, expressing gene *nadA* | This study |
| pTrc99a-*hypB* | pTrc99a derivate, expressing gene *hypB* | This study |
| pTrc99a-*rihA* | pTrc99a derivate, expressing gene *rihA* | This study |
| pCDF duet-*cadA-kdsA-nadA-hypB* | pCDF duet-*cadA* derivate, expressing gene *kdsA*, *nadA*, *hypB* | This study |

**Table S5.** Primers for experiments.

| Primers | Sequences (5′–3′) |
| --- | --- |
| cadR-F | AGATCTTCCCTAGTCTAGAAATAATTTTGTTTAACTTTAAGAAGGAGATATACC |
| cadR-R | TCTAGACTAGGGAAGATCTtccGGCATGTTCATCATCAGTAACCCGTATCGTGAGC |
| 28a-F | CCGCTCGAGCTAGCATAACCCCTTGGGG |
| 28a-R | CATGCCATGGTATATCTCCTTCTTAAAGTTAAAC |
| 99a-F | CATGCCATGGAATTCGAGCTCGG |
| 99a-R | CCGCTCGAGAAAAGGCCATCCGTCAG |
| gfp-F | TAAGAAGGAGATATACCATGGGCAGCAGCCATCATC |
| gfp-R | TTGCATGCCTGCAGGTCGACTTACTTGTACAGCTCGTCCATGCC |
| alaS-F | GATGATGAACATGCCGGATTTGGCGAAATTTTGCCGACTG |
| alaS-R | AAGTTAAACAAAATTATTAATTATCCTGAAATCAAGCTAACGAAATATC |
| ansB-F | CTGATGATGAACATGCCGGACTTTTTTATGCCGTTTAATTCTTCGTTTTG |
| ansB-R | TAAAGTTAAACAAAATTATTTTCATTCCTCCAGTTACGTGAACGC |
| ansP-F | CTGATGATGAACATGCCGGATTTTGCATCGTTTATCGATAACGGGTAGG |
| ansP-R | AAGTTAAACAAAATTATTTGCTCTCCCTGATTGCTTTAATG |
| asr-F | CTGATGATGAACATGCCGGATTATTATCAACGCTGTAATTTATTCAGCGTTTG |
| asr-R | TAAAGTTAAACAAAATTATTTGTCATACCCTCAATTTGTTTTTTCATTTAACC |
| bamE-F | CTGATGATGAACATGCCGGAAAACTTTTTTCCTGCTTCACGGTCAG |
| bamE-R | TAAAGTTAAACAAAATTATTAGTGATTTGATTCCTTTTCGGGCC |
| cadB-F | CTGATGATGAACATGCCGGATCACTTACAGAAACTTTTGTGTTATTTCACC |
| cadB-R | TAAAGTTAAACAAAATTATTGCTCTTCTCCTAATTTCATTTTTGAATTTGGAG |
| caiD-F | CTGATGATGAACATGCCGGACGTAATTGCTCGGGGAAAATAATTAGAAAG |
| caiD-R | TAAAGTTAAACAAAATTATTTCCTTTTCTCCATTTTTGCTTTTTCAGGG |
| cedA-F | TGATGAACATGCCGGACTCTCTAACTATAGACCACCAGCCAG |
| cedA-R | AAAGTTAAACAAAATTATTTACTGGCTTCACTAAACGCATATTAAAAATC |
| cspI-F | CTGATGATGAACATGCCGGAGTGTTCTGGTTTGTTACAAATTTATCTGAAGCAG |
| cspI-R | TAAAGTTAAACAAAATTATTAATTATTACCTTTTGAAGAAATTAGCCCTTGGG |
| csrA-F | CTGATGATGAACATGCCGGAGTATATCGGCTAAACTTAGGTTTAACAGAATGTAATGC |
| csrA-R | TAAAGTTAAACAAAATTATTTCTTTGCTCCTTGAAAGATTAAAAGAGTCGG |
| cyuA-F | TGATGAACATGCCGGACTGATCCCGGCGTGGC |
| cyuA-R | TTAAACAAAATTATTATTAAAAACCTTAAAATTTCAGGTAAATCAAGAAAATGCC |
| der-F | CTGATGATGAACATGCCGGAAAAGACGGAACCGTGTACTCTATTAC |
| der-R | TAAAGTTAAACAAAATTATTGTTTAAAGCCTCATTTTTATAAATCATCAGACAACG |
| efeU-F | CTGATGATGAACATGCCGGACCCTTGGCATTACCTCTCTTTGTTTAC |
| efeU-R | TAAAGTTAAACAAAATTATTGCCACCCACCCTTAAACGTTTTTC |
| fecC-F | GATGATGAACATGCCGGAGCTCTGGCAGATGTTAACCGCC |
| fecC-R | AAGTTAAACAAAATTATTTTCACAACGGTAAGCGGCTGATG |
| fimF-F | GATGATGAACATGCCGGATACCTCAGCGGAATGCCTTTAGCG |
| fimF-R | AAGTTAAACAAAATTATTCACGCCCCCTTAACGACATTC |
| fxsA-F | CTGATGATGAACATGCCGGATTGTGAAATAGATCACCGCTTTGGG |
| fxsA-R | TAAAGTTAAACAAAATTATTAGGTTTCTCCTGTAATAGCAGCCG |
| gnsB-F | CTGATGATGAACATGCCGGAATTCAGGAAAATTCTCTTTGTCTGTTTGTG |
| gnsB-R | TAAAGTTAAACAAAATTATTCATTTCCTCTTTTAGTCTGTTATGACTTTCCAG |
| insG-F | CTGATGATGAACATGCCGGATACTGATGTCACAAATTGAGCATATTTTTGTAGC |
| insG-R | TAAAGTTAAACAAAATTATTAAAAAAATCCGGAAACAAGTGAGCGTTTC |
| insH-F | CTGATGATGAACATGCCGGACGATTTGTTTGTCAAGATATTGTCC |
| insH-R | TAAAGTTAAACAAAATTATTATGAACCCTGTTCTATGGCTC |
| iscX-F | CTGATGATGAACATGCCGGATTTTGCATCGTTTATCGATAACGGGTAGG |
| iscX-R | TAAAGTTAAACAAAATTATTTGCTCTCCCTGATTGCTTTAATGAAAAAG |
| lepB-F | CTGATGATGAACATGCCGGAGGCGATATCAGCCGTAAGAAAAAGC |
| lepB-R | TAAAGTTAAACAAAATTATTGCCAACTCCTAAGGGTTATTTGTTG |
| lysO-F | CTGATGATGAACATGCCGGATAAAGCGCAAGAATATCTTTAAATGAAAACGAATACTC |
| lysO-R | TAAAGTTAAACAAAATTATTGAAAACCCAGTCCTTGCAAAGATGAAGTC |
| mreC-F | TGATGAACATGCCGGACCCGCTGACCTGTGTGG |
| mreC-R | GTTAAACAAAATTATTAAGTTATGCGTATTCTCGTATCAGACC |
| ompL-F | CTGATGATGAACATGCCGGATTGCATTGATTATTGAGGAAATCAACCAACG |
| ompL-R | TAAAGTTAAACAAAATTATTAATAAGTCCATTTTTTAAACGTAGGGAGGC |
| ompN-F | CTGATGATGAACATGCCGGATTATTATCATTTCAGAGGAATTATTTGATTAAGG |
| ompN-R | TAAAGTTAAACAAAATTATTTGAATAAATCCTTTAGTTATTTTATTTGCC |
| pqiC-F | CTGATGATGAACATGCCGGAAGCCTACAACAAGATGGTGGC |
| pqiC-R | TAAAGTTAAACAAAATTATTTGTTTCGCCCTCTTCGGCTC |
| pspB-F | CTGATGATGAACATGCCGGAATCGCTGGACGATCAGTTTGC |
| Pspb-R | TAAAGTTAAACAAAATTATTAAGTACTCCTTAGACGATGAGCGG |
| pyrF-F | CTGATGATGAACATGCCGGAAATGTTCCATTGTGCTCCGGC |
| pyrF-R | TAAAGTTAAACAAAATTATTGACCAGACCTTCTTGATGATGGGC |
| rluF-F | CTGATGATGAACATGCCGGACTGACATGGCTCACGATGTCG |
| rluF-R | TAAAGTTAAACAAAATTATTAAATTCTCCTGTAAAAAACGGAATATACCG |
| rph-F | CTGATGATGAACATGCCGGAGTAGGCGGTCATACTGCGGATC |
| rph-R | TAAAGTTAAACAAAATTATTATTGAAATCTCCGGCTTGAAACAAATGTG |
| rpiB-F | CTGATGATGAACATGCCGGAATGGTGAGTGTCTTTAACGCTATTGC |
| rpiB-R | TAAAGTTAAACAAAATTATTTCATCTTCCCCGTCCGTGCAAAAC |
| rrrQ-F | CTGATGATGAACATGCCGGATGAGGCAGCAGCCAGAAGG |
| rrrQ-Q | TAAAGTTAAACAAAATTATTAGATATTTATGATGAGGACGCTCGTGC |
| rseC-F | CTGATGATGAACATGCCGGATACGCCATCGAGCACCGATC |
| rseC-R | TAAAGTTAAACAAAATTATTTGCGCTGCCCCGAACTTAATATTC |
| symR-F | GATGATGAACATGCCGGAAAAACCGGCGGCTTCCAG |
| symR-R | AAGTTAAACAAAATTATTGACACGCATTCTATTGCACAACCGTTC |
| tdcF-F | TGATGAACATGCCGGACCGGAGCTGTGTGCAGG |
| tdcG-R | GTTAAACAAAATTATTATCCACACCCTCGGATTGCC |
| ybfG-F | CTGATGATGAACATGCCGGAGGTATTGGGTATCGGTTTATGCTTTG |
| ybfG-R | TAAAGTTAAACAAAATTATTCGCTACTTGCTCCTTGTGTGC |
| ybfK-F | CTGATGATGAACATGCCGGACACTAACCGCAATTTTTAATTTTGACATTTTTC |
| ybfK-R | TAAAGTTAAACAAAATTATTTCTGAAAAAAGCAATCTACCTGATTTATTTATCAG |
| ycjO-F | CTGATGATGAACATGCCGGACATGGGTGATGTGACGGGTTCTG |
| ycjO-R | TAAAGTTAAACAAAATTATTTGCGGATTCCTGTCCTGGGTTTAG |
| yddA-F | CTGATGATGAACATGCCGGAAGCAATATCCTTGATTATCAATAGCATAACC |
| yddA-R | TAAAGTTAAACAAAATTATTTAGCGAAAATTGATTGTGCCATCC |
| ydfJ-F | CTGATGATGAACATGCCGGAGGAGTTTTTTTGGGGCATATATGGG |
| ydfJ-R | TAAAGTTAAACAAAATTATTAATTCACAGGTGTTTTTTCCCATCC |
| ydiV-F | CTGATGATGAACATGCCGGATTTATAATCAGGATAATCTAAAAAAAGGACCCCTG |
| ydiV-R | TAAAGTTAAACAAAATTATTGGTAACCGTCCCTCTCGAAGG |
| yeaY-F | CTGATGATGAACATGCCGGACCGATTGCGTGTCAGATGTTTG |
| yeaY-R | TAAAGTTAAACAAAATTATTGGTGCGACTCCTTTTTTTCTCAGG |
| yedX-F | CTGATGATGAACATGCCGGAAATAAGTAGAATCTTCATGAAAATAATATGCC |
| yedX-R | TAAAGTTAAACAAAATTATTGTTTATATCCTTGTCATGTGAATGAGTTTTG |
| yfdF-F | CTGATGATGAACATGCCGGATCTGCATTTCCAGGATACTCc |
| yfdF-R | TAAAGTTAAACAAAATTATTGATTTTATTCCATTTTGATTGATTTGTTAC |
| yfeK-F | CTGATGATGAACATGCCGGACCTGCAGTGCCCTACTCTTATCG |
| yfeK-R | TAAAGTTAAACAAAATTATTTGAATTACCTTATCCTTGTTTTGTGATAGCCG |
| ylbI-F | CTGATGATGAACATGCCGGATGGTCTTTTACGATATCTGACATTGTGTAATAC |
| ylbI-R | TAAAGTTAAACAAAATTATTGGTTTATTTCCTCTTTCTTTCCCGGC |
| ypdK-F | CTGATGATGAACATGCCGGACGCCGTCTCGAGCAGTTTAG |
| ypdK_R | TAAAGTTAAACAAAATTATTTTTACATCTCTCCGCGTAATTACTGTTACG |
| yqeG-F | CTGATGATGAACATGCCGGAGCAGCAGTCAGAGACATAACTGG |
| yqeG-R | TAAAGTTAAACAAAATTATTTATCTGAACGTATCATCTACAAATTAAACAAAATGG |
| yqiK-F | CTGATGATGAACATGCCGGATTATTTACTACTGGAGCCTGAAGAAGG |
| yqiK-R | TAAAGTTAAACAAAATTATTTATTAAATTTCCTTTTGATTAATAAATTACAATATTTGGGGC |
| add-F | CTGATGATGAACATGCCGGAAAAAAGGTGTGGCTGGATTAATTAAC |
| add-R | TAAAGTTAAACAAAATTATTGGTCGCACTCTTTTTCTGG |
| aldA-F | CTGATGATGAACATGCCGGATGACTTTTATTTCTGCCTTTTATTCC |
| aldA-R | TAAAGTTAAACAAAATTATTGGCGACTCCTGTGATTTATATG |
| asnA-F | CTGATGATGAACATGCCGGAGGATTTTCTTATGCGGATTGATGATTC |
| asnA-R | TAAAGTTAAACAAAATTATTTTTTTATACTCCTGCGTCCTGTTG |
| aspA-F | CTGATGATGAACATGCCGGACGTACAGACCATTAAAGCAGTGTAG |
| aspA-R | TAAAGTTAAACAAAATTATTAGTGTATTACCTTTAATAATTAAGTGTGCC |
| cueO-F | CTGATGATGAACATGCCGGACTTAAAGAAGATAAACGTTAGCACACTG |
| cueO-R | TAAAGTTAAACAAAATTATTAGTTATTTCCTTATTCTTAAGCAGGCG |
| cyoD-F | TGATGAACATGCCGGACTGGATGGCGGTGCTGATG |
| cyoD-R | GTTAAACAAAATTATTTACATCGCCCCCATCAGATAAACAACAGtg |
| dsbD-F | CTGATGATGAACATGCCGGAGCTGGAGCAAGAATACGAAGTGC |
| dsbD-R | TAAAGTTAAACAAAATTATTGAGAGGTAATCTGTGTCTCCGTGTG |
| gapA-F | CTGATGATGAACATGCCGGACAAAGGAAGAGTGAGGCGAG |
| gapA-R | TAAAGTTAAACAAAATTATTATATTCCACCAGCTATTTGTTAGTG |
| gldA-F | CTGATGATGAACATGCCGGAACTGTTTACTGGCAGGATGTG |
| gldA-R | TAAAGTTAAACAAAATTATTAATTGCTCCTTTAGAGATGAGTAGTG |
| grcA-F | CTGATGATGAACATGCCGGATTGCGACCATACTTGATGTGTG |
| grcA-R | TTAAACAAAATTATTGTGATGCCTCCCCGG |
| hupA-F | GATGAACATGCCGGACCTGGAGTCTTCCCTTTCGC |
| hupA-R | TTAAACAAAATTATTAAGTTATCCTTACAATGTGTTTATCGCTTGC |
| hybB-R | TTAAACAAAATTATTCGCTCACTCCTGGTCTATTTC |
| hypB-F | GATGAACATGCCGGAGCCGAATGCTGGTGTGAAAC |
| ilvB-F | GATGAACATGCCGGAGTGGTCGTCGTGCGTGTG |
| ilvB-R | TTAAACAAAATTATTGCTCCAGTCCTTTTCTTCTGGG |
| ilvN-F | CTGATGATGAACATGCCGGAAATAACGAAGCCGATCCGCAG |
| ilvN-R | TAAAGTTAAACAAAATTATTGGCTTATTCCCCCACCATTTCAG |
| leuA-F | CTGATGATGAACATGCCGGAACTACTACTAAACGCATCTTCTTTGCG |
| leuA-R | TAAAGTTAAACAAAATTATTGGTTTGGGTCCTTGTCTCTTTTAGAG |
| leuL-F | CTGATGATGAACATGCCGGATATCGCTTACTTTAACCACCGCAG |
| leuL-R | TAAAGTTAAACAAAATTATTTAAATCAGCTCCAGATGAATGCGATATGC |
| lldD-F | TGATGAACATGCCGGACGGTGATGCTGACGGG |
| lldD-R | GTTAAACAAAATTATTGCGTTTTTCTCCCTCGAATGC |
| lldR-F | CTGATGATGAACATGCCGGAATCTATCGCTATCGCCTGTGCG |
| lldR-R | TAAAGTTAAACAAAATTATTTAAGGAATCATCCACGTTAAGACATAAGCC |
| ltdC-F | GATGATGAACATGCCGGACGCTTAATGGTAAATCGGGGGC |
| ltdC-R | AAGTTAAACAAAATTATTCAAAAATCCATAATAAATATAAGGTTATTGTAATAAACAG |
| moaA-F | GATGATGAACATGCCGGATTACACGCTAGTATCGGCATAACC |
| moaA-R | AAGTTAAACAAAATTATTGTACACCTTTCCAGATACGGGAG |
| nrdD-F | CTGATGATGAACATGCCGGAAATTGCTACTACAGCTCCCCAC |
| nrdD-R | TAAAGTTAAACAAAATTATTGATCGCTCCATATTTTTAAGAACAGG |
| nuoG-F | CTGATGATGAACATGCCGGAATTTCCGCGAAGAATTTGAGGC |
| nuoG-R | TAAAGTTAAACAAAATTATTTAGCATGCTTCCAGTTTTCTCAGTCAG |
| pflB-F | GATGATGAACATGCCGGACTGATTCCGGTTACGATCG |
| pflB-R | AAGTTAAACAAAATTATTGTAACACCTACCTTCTTAAGTGG |
| pheA-F | CTGATGATGAACATGCCGGAAAACATGAAA CACATACCGTTTTTCTTC |
| pheA-R | TAAAGTTAAACAAAATTATTAGTGTTGCCTTTTTGTTATCAATAAAAAAG |
| phnC-F | CTGATGATGAACATGCCGGACAACAACCAACGCAATAACCCG |
| phnC-R | TAAAGTTAAACAAAATTATTGATGCAGCCTCCCTAAAAAAGTGG |
| phnD-F | GATGAACATGCCGGACGCATCGTCGCCCTGC |
| phnD-R | GTTAAACAAAATTATTTCGTTTTCCTCAATGATGGGGATG |
| phnE-F | CTGATGATGAACATGCCGGAGCAAAAACCACCGAGATTCAGGC |
| phnE-R | TAAAGTTAAACAAAATTATTCAACGCCTGCACATTGTCGG |
| phnG-F | GATGATGAACATGCCGGAGCCAAAGAGTGCCAGCGTC |
| phnG-R | AAGTTAAACAAAATTATTTCAGTGCTCCATAGTGAATTCAATCATGTC |
| phnI-F | TGATGAACATGCCGGAAGAACGAATGATCGCTCCGC |
| phnI-R | GTTAAACAAAATTATTCAGCACACCTCCACATGAGTG |
| phnJ-F | GATGAACATGCCGGACGACAGGCCCGGCG |
| phnJ-R | TTAAACAAAATTATTGGTTCTGCTCCTGTTGCAGAC |
| phnL-F | CTGATGATGAACATGCCGGAGTATACACAGCTGCTGGTGTCATC |
| phnL-R | TAAAGTTAAACAAAATTATTTTTTCGCCTCGTGGTTCGGG |
| phoU-F | GATGATGAACATGCCGGAGCAGGCTGCGCGTTGTTC |
| phoU-R | AAGTTAAACAAAATTATTAACGCACTCCTGAATCAACCGTAAC |
| psiF-F | CTGATGATGAACATGCCGGAACACCATGAAAGCCGCTCTG |
| psiF-R | TAAAGTTAAACAAAATTATTAGCACCATCCCTCTTCATGTTTTAAC |
| pstB-F | TGATGAACATGCCGGACCTCTCCCCAGAGGGGCG |
| pstB-R | GTTAAACAAAATTATTTGCAATCTTTTCCTCATCGTGCCTG |
| pykA-F | CTGATGATGAACATGCCGGATTAATTTAAGTGACGATCGCTAAAAACGAC |
| pykA-R | TAAAGTTAAACAAAATTATTGTAATACTCCGTTGACTGAAACAACC |
| rbsA-F | CTGATGATGAACATGCCGGAAACACCAGGGAAATACCATTGAAATTCG |
| rbsA-R | AAGTTAAACAAAATTATTGACGGCCTCAGAACGTCACG |
| rpmF-F | GATGATGAACATGCCGGATGTGAAGTGTCCGAAGCGGAC |
| rpm-R | AAGTTAAACAAAATTATTGGACCTTACTCCTCAATTACGGTTTATCC |
| sdhA-F | CTGATGATGAACATGCCGGATTCTATCTTGATCCATGCCTGGATCGG |
| sdhA-R | AAGTTAAACAAAATTATTCACACACCCCACACCACAAC |
| sdhB-F | CTGATGATGAACATGCCGGATTCCCGGATCGTGATGATGAAAACTG |
| sdhB-R | TAAAGTTAAACAAAATTATTTTTCCTGTCTCCGCATTAGTAAGTACG |
| sdhD-F | TGATGAACATGCCGGATACCGCTCTGGCGTATCACG |
| sdhD-R | GTTAAACAAAATTATTACGAGGACTCCTGCGAGAAG |
| soxS-F | GATGAACATGCCGGACCGCTGCGTTTCGCCAC |
| soxS-R | TTAAACAAAATTATTAAATCTGCCTCTTTTCAGTGTTCAGTTCG |
| sucA-F | CTGATGATGAACATGCCGGAAACTGCCCCTGACACTAAGACAG |
| sucA-R | TAAAGTTAAACAAAATTATTCGTGATCCCTTAAGCATCTTTTTTATGC |
| sucB-F | CTGATGATGAACATGCCGGACCGTGAAGTGATTCCGTTTGGG |
| sucB-R | TAAAGTTAAACAAAATTATTTGTGTATCCTTTATTTATTCGACGTTCAGCGC |
| ygiB-F | GATGATGAACATGCCGGATGATGACGACGACGGGGC |
| ygiB-R | AAGTTAAACAAAATTATTTTTTGTCTTCCGGGACCAGTGG |
| yhaL-F | CTGATGATGAACATGCCGGACGCTTACGCATCATGAAGAGAAAGC |
| yhaL-R | TAAAGTTAAACAAAATTATTCATCATCTCCCTGTAAAAACAACTAGAC |
| yjhD-F | GATGATGAACATGCCGGACAATCCTTTATCTACGCCGCCC |
| yjhD-R | AAGTTAAACAAAATTATTAGTGATGCTCCTTACTCTTGAGACAG |
| yoaE-F | CTGATGATGAACATGCCGGACAGTATGGATCGTCACCGACGTC |
| yoaE-R | TAAAGTTAAACAAAATTATTGACAAGCTCCTGCTTAAGGAATAGC |
| zraP-F | CTGATGATGAACATGCCGGATAAGTTTCAATGAGTAAAAATGACTCGCTACCCG |
| zraP-R | TAAAGTTAAACAAAATTATTGGTTAATCCTCCAGTGGTTGTCTTACTTC |
| pyrF-10 | GAAGATCTTGTGCTCCGGCAACGA |
| pyrF-20 | GAAGATCTCAACGACGGCGCAG |
| pyrF-30 | GAAGATCTGCAGAAAAAGCCTGCCAGG |
| pyrF-40 | GAAGATCTCCTGCCAGGGGAGAAATC |
| pyrF-50 | GAAGATCTGAGAAATCGCAACTGTTAATTTTTTATTTCC |
| pyrF-60 | GAAGATCTAACTGTTAATTTTTTATTTCCACGGGTAGA |
| pyrF-70 | GAAGATCTTTAATTTTTTATTTCCACGGGTAGAATGC |
| pyrF-xia | GCTCTAGAGACCAGACCTTCTTGATGATGG |
| pyrF-xia -10 | GCTCTAGATCTTGATGATGGGCACCG |
| pyrF-xia -20 | GCTCTAGAGGGCACCGGAAGTGG |
| pyrF-xia -30 | GCTCTAGAAGTGGCGCGAAACAGG |
| pyrF-xia -40 | GCTCTAGAAACAGGTAAACGGCGAGC |
| pyrF-xia -50 | GCTCTAGACGGCGAGCATTCTACCC |
| pyrF-xia -60 | GCTCTAGAATTCTACCCGTGGAAATAAAAAATTAACAG |
| pyrF-shang | GAAGATCTAATGTTCCATTGTGCTCCG |
| *kdsA*-F | ACAGACCATGGAATTCATGAAACAAAAAGTGGTTAGCATTGGC |
| *kdsA*-R | CGACTCTAGAGGATCCTTACTTGCTGGTATCCAGTTCTTCGAAAC |
| *nadA*-F | ACAGACCATGGAATTCATGAGCGTAATGTTTGATCCAGACAC |
| *nadA*-R | CGACTCTAGAGGATCCTTATCCACGTAGTGTAGCCGCAAAAT |
| *hypB*-F | ACAGACCATGGAATTCATGTGTACAACATGCGGTTGC |
| *hypB*-R | CGACTCTAGAGGATCCCTATGCACATCGCTGTGTCTC |
| *rihA*-F | ACAGACCATGGAATTCATGGCACTGCCAATTCTGTTAGA |
| *rihA*-R | CGACTCTAGAGGATCCTTAAGCGTAAAATTTCAGACGATCAGCC |
| P-I-F | ACTGTCAGTCAGTCAGTCGATCGAGTAGCACCAGCAGGACTGATCACTGCACCCTTTCGTCTTCACCTCGAG |
| P-I-R | CCAGCTCTGCGTCAGTCGCAGTACGCTGTCAGTCGTCAGCGAGCTGACGATTTAGCGGCCGCATAGGCC |
| P-II-F | ATCGTCAGCTCGCTGACGACTGACAGCGTACTGCGACTGACGCAGAGCTGGCACACAGAATTCATTAAAGAGGAGAAATTAACTATG |
| P-II-R | TTGCGACGCATACGTCGCAACTGACTCGACAGCTGACTCATCACATCTGCATTAGCGGCCGCATAGGCC |
| P-III-F | TGCAGATGTGATGAGTCAGCTGTCGAGTCAGTTGCGACGTATGCGTCGCAACACACAGAATTCATTAAAGAGGAGAAATTAACTATG |
| P-III-R | CAAGTAGGCGTCGTTCAGCTTCCAGTACGACTTACGTGCAAGGCATGCAGTTTAGCGGCCGCATAGGCC |
| M-F | AACTCAGAAGTGAAACGCCGTAG |
| M-R | CGTGCAGTCGATGATAAGCTGTCAA |

**References**

[1]	Y. Zhang, L. Chen, Y. M. He, W. Y. Luo, K. X. Li, Y. G. Min, *Polymers-Basel* **2023**, *15*, 1088.

[2]	A. Elgowainy, J. Han, H. Cai, M. Wang, G. S. Forman, V. B. DiVita, *Environ Sci Technol* **2014**, *48*, 7612.

[3]	V. Froidevaux, C. Negrell, S. Caillol, J. P. Pascault, B. Boutevin, *Chem Rev* **2016**, *116*, 14181.

[4]	M. Ghasemlou, F. Daver, E. P. Ivanova, B. Adhikari, *Eur Polym J* **2019**, *118*, 668.

[5]	L. X. Fang, X. Y. Hao, J. Fan, X. L. Liu, Y. R. Chen, L. Wang, X. Y. Huang, H. Song, Y. X. Cao, *Nat Commun* **2025**, *16*, 3060.

[6]	L. X. Fang, X. L. Liu, Z. X. Chen, J. Q. Zhang, L. Wang, Y. X. Cao, *Metab Eng* **2025**, *92*, 13.

[7]	Y. Y. Gao, W. F. Peng, J. A. Wei, D. C. Guo, Y. J. Zhang, Q. Q. Yu, C. Wang, L. G. Wang, *Acs Appl Mater Inter* **2024**, *16*, 48005.

[8]	S. Kind, S. Neubauer, J. Becker, M. Yamamoto, M. Volkert, G. von Abendroth, O. Zelder, C. Wittmann, *Metab Eng* **2014**, *25*, 113.

[9]	M. O. Lee, J. H. Kim, J. Park, S. Y. Kim, *Green Chem* **2021**, *23*, 6469.

[10]	H. T. Kim, K. A. Baritugo, Y. H. Oh, S. M. Hyun, T. U. Khang, K. H. Kang, S. H. Jung, B. K. Song, K. Park, I. K. Kim, M. O. Lee, Y. Kam, Y. T. Hwang, S. J. Park, J. C. Joe, *Acs Sustain Chem Eng* **2018**, *6*, 5296.

[11]	C. H. Tseng, P. S. Tsai, *Polymers-Basel* **2022**, *14*, 3879.

[12]	J. Feng, Y. Han, S. Xu, Y. Liao, Y. T. Wang, S. Xu, H. Li, X. Wang, K. Q. Chen, *Bioresource Technol* **2024**, *398, 130529*.

[13]	V. F. Wendisch, *Metab Eng* **2020**, *58*, 17.

[14]	M. Pisani, P. Carbonell, *Acs Synth Biol* **2025**, *14*, 3267.

[15]	C. Y. Li, T. Jiang, M. Li, Y. S. Zou, Y. J. Yan, *Biotechnol Adv* **2022**, *54*, 107853.

[16]	L. Qin, X. Liu, K. Xu, C. Li, *Curr Opin Biotech* **2022**, *75*, 102694.

[17]	X. F. Chen, X. X. Xia, S. Y. Lee, Z. G. Qian, *Biotechnol Bioeng* **2018**, *115*, 1014.

[18]	M. R. Lu, Y. Y. Sha, V. Kumar, Z. X. Xu, R. Zhai, M. J. Jin, *Biotechnol Adv* **2024**, *72*, 108339.

[19]	F. Yu, C. Y. Li, T. Zhang, J. W. Zhou, J. H. Li, J. Chen, G. C. Du, X. R. Zhao, *Metab Eng* **2024**, *84*, 59.

[20]	Q. Guo, Y. F. Zhong, X. Y. Chen, Y. W. Li, Y. X. Yang, Z. K. Nie, T. Q. Shi, *J Agr Food Chem* **2025**, *73*, 8440.

[21]	L. Qin, S. X. Dong, J. Yu, X. Y. Ning, K. Xu, S. J. Zhang, L. Xu, B. Z. Li, J. Li, Y. J. Yuan, C. Li, *Metab Eng* **2020**, *61*, 160.

[22]	C. Acierno, F. Barletta, R. Nevola, L. Rinaldi, F. C. Sasso, L. E. Adinolfi, A. Caturano, *Int J Mol Sci* **2025**, *26*, 5574.

[23]	X. Wang, X. Guo, J. Wang, H. Li, F. He, S. Xu, K. Q. Chen, P. K. Ouyang, *Syn Syst Biotechno* **2021**, *6*, 243.

[24]	V. Solovyev, A. Salamov, **2011**, "Chapter 4-Automatic annotation of microbial genomes and metagenomic sequences." in *Metagenomics and its applications in agriculture, biomedicine and environmental studies*, Edited by R. W. Li, Nova Science Publishers, ISBN 978-1-61668-682-6.

[25]	J. C. Hu, C. A. Gross, *Mol Gen Genet* **1983**, *191*, 492.

[26]	T. Shimada, Y. Yamazaki, K. Tanaka, A. Ishihama, *Plos One* **2014**, *9*, e90447.

[27]	S. S. Chen, M. Hu, A. Q. Hu, Y. Xue, S. Wang, F. Liu, C. H. Li, X. F. Zhou, J. N. Zhou, *Mol Plant Pathol* **2022**, *23*, 1487.

[28]	H. K. Bi, C. Y. Zhang, *Curr Microbiol* **2014**, *69*, 218.

[29]	H. A. Nash, C. A. Robertson, E. Flamm, R. A. Weisberg, H. I. Miller, *J Bacteriol* **1987**, *169*, 4124.

[30]	L. M. O. Monteiro, A. Sanches-Medeiros, C. A. Westmann, R. Silva-Rocha, *Front Bioeng Biotech* **2020**, *8*, 510.

[31]	S. Reverchon, S. Meyer, R. Forquet, F. Hommais, G. Muskhelishvili, W. Nasser, *Nucleic Acids Res* **2021**, *49*, 776.

[32]	J. S. Gao, M. H. Du, J. H. Zhao, J. H. Zhao, N. Xu, H. M. Du, J. S. Ju, L. Wei, J. Liu, *Metab Eng* **2022**, *73*, 144.

[33]	W. Soksawatmaekhin, A. Kuraishi, K. Sakata, K. Kashiwagi, K. Igarashi, *Mol Microbiol* **2004**, *51*, 1401.

[34]	H. Samartzidou, A. H. Delcour, *J Bacteriol* **1999**, *181*, 791.

[35]	S. Shi, Y. W. Choi, H. M. Zhao, M. H. Tan, E. L. Ang, *Bioresource Technol* **2017**, *245*, 1343.

[36]	H. T. Hu, R. R. Sun, Q. X. Zhai, W. Zhao, R. J. Yang, X. Lyu, *J Agr Food Chem* **2026**, *74*, 10375.

[37]	L. Qin, B. Yang, R. Huang, H. C. Zhang, Q. Y. Li, X. Y. Li, Y. Q. Tan, W. T. Sun, H. Y. Jia, B. Hu, B. Lv, C. Li, *Nat Commun* **2026**, *17*, 5081.

[38]	Y. Wu, R. Y. Zhu, L. A. Mitchell, L. Ma, R. Liu, M. Zhao, B. Jia, H. Xu, Y. X. Li, Z. M. Yang, Y. Ma, X. Li, H. Liu, D. Liu, W. H. Xiao, X. Zhou, B. Z. Li, Y. J. Yuan, J. D. Boeke, *Nat Commun* **2018**, *9*, 1935.

[39]	S. Centeno-Leija, J. Utrilla, N. Flores, A. Rodriguez, G. Gosset, A. Martinez, *Anton Leeuw Int J G* **2013**, *104*, 913.

[40]	R. M. Liu, L. Y. Liang, E. F. Freed, A. Choudhury, C. A. Eckert, R. T. Gill, *Nat Commun* **2020**, *11*, 4050.

[41]	W. Abou-Jaoudé, P. Traynard, P. Monteiro, J. Saez-Rodriguez, T. Helikar, D. Thieffry, C. Chaouiya, *Front Genet* **2016**, *7*, 94.

[42]	A. Pandey, P. Sharma, D. Mishra, S. Dey, R. Malviya, D. Gayen, *Int J Biol Macromol* **2023**, *234*, 123757.

[43]	H. Fujishima, A. Nishimura, M. Wachi, H. Takagi, T. Hirasawa, H. Teraoka, K. Nishimori, T. Kawabata, K. Nishikawa, K. Nagai, *Microbiol-Sgm* **2002**, *148*, 103.

[44]	G. Klein, S. Raina, *Int J Mol Sci* **2019**, *20*, 2.

[45]	Q. R. Wang, H. Kim, T. M. Halvorsen, S. J. Chen, C. S. Hayes, C. R. Buie, *Front Bioeng Biotech* **2023**, *11*, 991784.

[46]	Y. J. Zhou, L. Wang, F. Yang, X. P. Lin, S. F. Zhang, Z. B. K. Zhao, *Appl Environ Microb* **2011**, *77*, 6133.

[47]	T. Maier, A. Bock, *Biochemistry-Us* **1996**, *35*, 10089.

[48]	F. Cai, T. T. Ngu, H. Kaluarachchi, D. B. Zamble, *J Biol Inorg Chem* **2011**, *16*, 857.

[49]	M. R. Leach, S. Sandal, H. W. Sun, D. B. Zamble, *Biochemistry-Us* **2005**, *44*, 12229.

[50]	J. H. Yang, Z. Peng, Q. Zhu, J. Zhang, G. C. Du, *Acs Synth Biol* **2022**, *11*, 1521.

[51]	B. Arivett, M. Farone, R. Masiragani, A. Burden, S. Judge, A. Osinloye, C. Minici, M. Degano, M. Robinson, P. Kline, *Bba-Proteins Proteom* **2014**, *1844*, 656.

[52]	C. Petersen, L. B. Moller, *J Biol Chem* **2001**, *276*, 884.

[53]	X. L. Chen, L. Y. Wang, V. Sourjik, *Curr Opin Microbiol* **2025**, *87*, 102632.

[54]	Y. Itoh, J. D. Rice, C. Goller, A. Pannuri, J. Taylor, J. Meisner, T. J. Beveridge, J. F. Preston, T. Romeo, *J Bacteriol* **2008**, *190*, 3670.

[55]	C. de la Fuente-Núñez, F. Reffuveille, L. Fernández, R. E. W. Hancock, *Curr Opin Microbiol* **2013**, *16*, 580.

[56]	Y. W. Zhang, X. M. Sun, Q. Z. Wang, J. Q. Xu, F. Dong, S. Q. Yang, J. W. Yang, Z. X. Zhang, Y. Qian, J. Chen, J. Zhang, Y. M. Liu, R. S. Tao, Y. Jiang, J. J. Yang, S. Yang, *Acs Synth Biol* **2020**, *9*, 1998.

[57]	S. Y. Gao, J. C. Lu, T. T. Wang, S. Xu, X. Wang, K. Q. Chen, P. K. Ouyang, *Microb Cell Fact* **2022**, *21*, 248.

[58]	Y. Wang, X. F. Cen, G. P. Zhao, J. Wang, *J Bacteriol* **2012**, *194*, 5237.

[59]	L. Wei, N. Xu, Y. R. Wang, W. Zhou, G. Q. Han, Y. H. Ma, J. Liu, *Appl Microbiol Biot* **2018**, *102*, 4117.

[60]	M. Kitagawa, T. Ara, M. Arifuzzaman, T. Ioka-Nakamichi, E. Inamoto, H. Toyonaga, H. Mori, *DNA Res* **2005**, *12*, 291.

[61]	G. N. Vemuri, M. A. Eiteman, E. Altman, *J Ind Microbiol Biot* **2002**, *28*, 325.

[62]	W. C. Ma, W. J. Cao, H. Zhang, K. Q. Chen, Y. Li, P. K. Ouyang, *Biotechnol Lett* **2015**, *37*, 799.

[63] J. Kalinowski, J. Cremer, B. Bachmann, L. Eggeling, H. Sahm, A. Puhler, *Mol. Microbiol.* **1991**, *5*, 1197.

[64] W. J. Wu, Y. Zhang, D. H. Liu, Z. Chen, *Metab. Eng.* **2019**, *52*, 77.

[65] Z. G. Qian, X. X. Xia, S. Y. Lee, *Biotechnol. Bioeng.* **2011**, *108*, 93.

[66] T. Mimitsuka, H. Sawai, M. Hatsu, K. Yamada, *Biosci. Biotech. Bioch.* **2007**, *71*, 2130.
